# Conditional mutagenesis *in vivo* reveals cell type- and infection stage-specific requirements for LANA in chronic MHV68 infection

**DOI:** 10.1371/journal.ppat.1006865

**Published:** 2018-01-24

**Authors:** Eduardo Salinas, Arundhati Gupta, Jeffrey M. Sifford, Darby G. Oldenburg, Douglas W. White, J. Craig Forrest

**Affiliations:** 1 Department of Microbiology and Immunology and Center for Microbial Pathogenesis and Host Inflammatory Responses, University of Arkansas for Medical Sciences, Little Rock, Arkansas, United States of America; 2 Gundersen Health System, La Crosse, Wisconsin, United States of America; Harvard University, UNITED STATES

## Abstract

Gammaherpesvirus (GHV) pathogenesis is a complex process that involves productive viral replication, dissemination to tissues that harbor lifelong latent infection, and reactivation from latency back into a productive replication cycle. Traditional loss-of-function mutagenesis approaches in mice using murine gammaherpesvirus 68 (MHV68), a model that allows for examination of GHV pathogenesis *in vivo*, have been invaluable for defining requirements for specific viral gene products in GHV infection. But these approaches are insufficient to fully reveal how viral gene products contribute when the encoded protein facilitates multiple processes in the infectious cycle and when these functions vary over time and from one host tissue to another. To address this complexity, we developed an MHV68 genetic platform that enables cell-type-specific and inducible viral gene deletion *in vivo*. We employed this system to re-evaluate functions of the MHV68 latency-associated nuclear antigen (mLANA), a protein with roles in both viral replication and latency. Cre-mediated deletion in mice of *loxP*-flanked *ORF73* demonstrated the necessity of mLANA in B cells for MHV68 latency establishment. Impaired latency during the transition from draining lymph nodes to blood following mLANA deletion also was observed, supporting the hypothesis that B cells are a major conduit for viral dissemination. Ablation of mLANA in infected germinal center (GC) B cells severely impaired viral latency, indicating the importance of viral passage through the GC for latency establishment. Finally, induced ablation of mLANA during latency resulted in complete loss of affected viral genomes, indicating that mLANA is critically important for maintenance of viral genomes during stable latency. Collectively, these experiments provide new insights into LANA homolog functions in GHV colonization of the host and highlight the potential of a new MHV68 genetic platform to foster a more complete understanding of viral gene functions at discrete stages of GHV pathogenesis.

## Author summary

Gammaherpesviruses (GHVs), including the human pathogens Epstein-Barr virus and Kaposi sarcoma-associated herpesvirus, establish lifelong infections that can lead to cancer. Defining the functions of viral gene products in acute replication and chronic infection is important for understanding how these viruses cause disease. Infection of mice with the related GHV, murine gammaherpesvirus 68 (MHV68), provides a tractable small animal model for defining how viral gene products function in chronic infection and understanding how host factors limit disease. Here we describe the development of a new viral genetic platform that enables the targeted deletion of specific genes from the viral genome in discrete host cells or at distinct times during infection. We utilize this system to better define requirements for the conserved latency-associated nuclear antigen in MHV68 lytic replication and latency in mice. This work highlights the utility of this MHV68 genetic platform for defining mechanisms of GHV infection and disease.

## Introduction

Gammaherpesviruses (GHVs) are large, enveloped, double-strand DNA viruses that include the human pathogens Epstein-Barr virus (EBV) and Kaposi sarcoma-associated herpesvirus (KSHV). GHVs establish lifelong, chronic infections in their hosts and are responsible for causing lymphoproliferative disorders (LPD) and cancer [1]. Like other herpesvirus subfamilies, infections by GHVs are characterized by two distinct stages: the productive or lytic replication cycle and latency. The lytic phase of infection involves expression of all kinetic classes (immediate-early, early, and late) of viral genes, production of infectious progeny, and viral dissemination, provoking inflammation and a potent antiviral immune response [2–4]. In contrast, latency is characterized by the absence of readily detectable infectious virion production and minimal viral gene expression, allowing infected cells to evade the immune system. Establishment and maintenance of latency enables GHVs to persist indefinitely within the host [1,2,4]. Given a proper stimulus, latent GHVs can undergo reactivation, a re-entry into the lytic cycle, which likely serves as a mechanism for re-seeding latent reservoirs and infecting new hosts [3,4]. Understanding the intricacies of GHV infection is imperative to deciphering the mechanisms that drive GHV disease.

Murine gammaherpesvirus 68 (MHV68) is a rodent GHV that is genetically and phenotypically related to EBV and KSHV [1,3,4]. MHV68 replicates efficiently in cell culture and readily infects and establishes latency in inbred and outbred strains of mice. MHV68 tropism for cells in the infected animal, such as B cells during latency, is similar to human GHVs. Since both the virus and host are amenable to targeted genetic manipulation, MHV68 infection of mice provides a versatile small animal model for studying the functions of viral gene products in GHV infection and for defining host responses to infection [1,3].

Though the sequence of events is not completely defined, MHV68 is thought to follow a complex route of dissemination in order to colonize a host. Acute replication in airway alveolar epithelial cells is thought to seed CD11c^+^ dendritic cells, which traffic virus from the primary site of infection to draining lymph nodes [5]. Studies in which the MHV68 non-coding RNA TMER4 was disrupted suggest that trafficking from draining lymph nodes to the spleen occurs hematogenously [6]. Although latency is established in splenic macrophages after IP inoculation of B cell-deficient MuMT mice, latency is not established in the spleens of these mice after IN inoculation [7,8]. Given that adoptive transfer of B cells into MuMT mice overcomes this defect [9], B cells appear to be required for establishment of splenic latency following IN inoculation and one model posits that B cells facilitate viral transit from draining LNs to the spleen via the blood. However, this has not been directly demonstrated. Considerably less is known about specific viral determinants that control viral mobility and tropism across different tissues during infection. One viral gene that potentially facilitates dissemination is the latency-associated nuclear antigen (LANA).

LANA is a conserved protein encoded by the *ORF73* gene in members of the *Rhadinovirus* genus of GHVs [10,11]. LANA is expressed as an immediate-early gene product during the lytic phase of infection and is also one of the few viral genes expressed during latency [12–15]. Numerous functions are described for LANA homologs in tissue culture systems, including transcriptional regulation of viral and host genes, inhibition of tumor suppressors and cell-cycle dysregulation, regulation of viral DNA replication during latency, and maintenance of the latent viral episome for long-term persistence [4,10,16]. Studies using an mLANA-null MHV68 (73.STOP) defined functions for LANA *in vivo* in promoting efficient acute replication, establishment of latency, and reactivation from latency [17–23]. However, an inherent problem with this approach is that the absence of mLANA at early stages of infection may indirectly influence phenotypes observed at subsequent stages. For example, productive replication of mLANA-null MHV68 in the lungs of mice is significantly impaired, which may reduce the number of virus-seeded B cells capable of trafficking to the spleen [19]. Consequently, this may indirectly reduce the number of latently infected splenocytes and thereby obfuscate identification of the function of mLANA in facilitating splenic latency.

To address this limitation of prior studies, we have developed a genetic platform that enables dissection of mLANA functions at discrete steps in the MHV68 infectious cycle. We engineered a recombinant MHV68 that contains *loxP* sites flanking *ORF73* (O73.loxP MHV68) to enable cell-type specific and inducible ablation of mLANA. We use this system to define requirements for mLANA in B cells, and in particular germinal center B cells, in colonization of the host by MHV68. We further test, by inducible deletion *after* the establishment of latency, the requirement of mLANA for maintenance of viral genomes during chronic MHV68 infection. Through these studies we delineate cell-type specific and temporal functions of mLANA in the establishment and maintenance of MHV68 latency and establish a more nuanced virus-host genetic approach to more precisely understand chronic GHV infection.

## Results

### Development and validation of a genetic platform for conditional viral gene deletion

LANA homologs are multi-functional proteins that play roles in lytic replication, latency, and reactivation from latency [17–24]. In order to define roles for MHV68 LANA at discrete steps in the infection cycle, we developed a genetic platform to enable inducible deletion of viral genes *in vivo* in infected cells that express Cre recombinase. We first generated a new MHV68 BAC (termed FRT BAC) in which the *loxP* sites that flank the non-viral BAC vector sequences [25] were replaced with *FRT* sites (**S1 Fig**). This allowed us to make use of *loxP* sequences elsewhere in the viral genome while preserving the ability to excise the non-viral BAC vector sequences using Flp recombinase (**S2 Fig**). MHV68 produced using the newly derived BAC exhibits WT levels of acute replication, latency establishment, and reactivation from latency when compared directly to virus derived from the original MHV68 BAC (**S3 Fig**).

**Fig 1:**
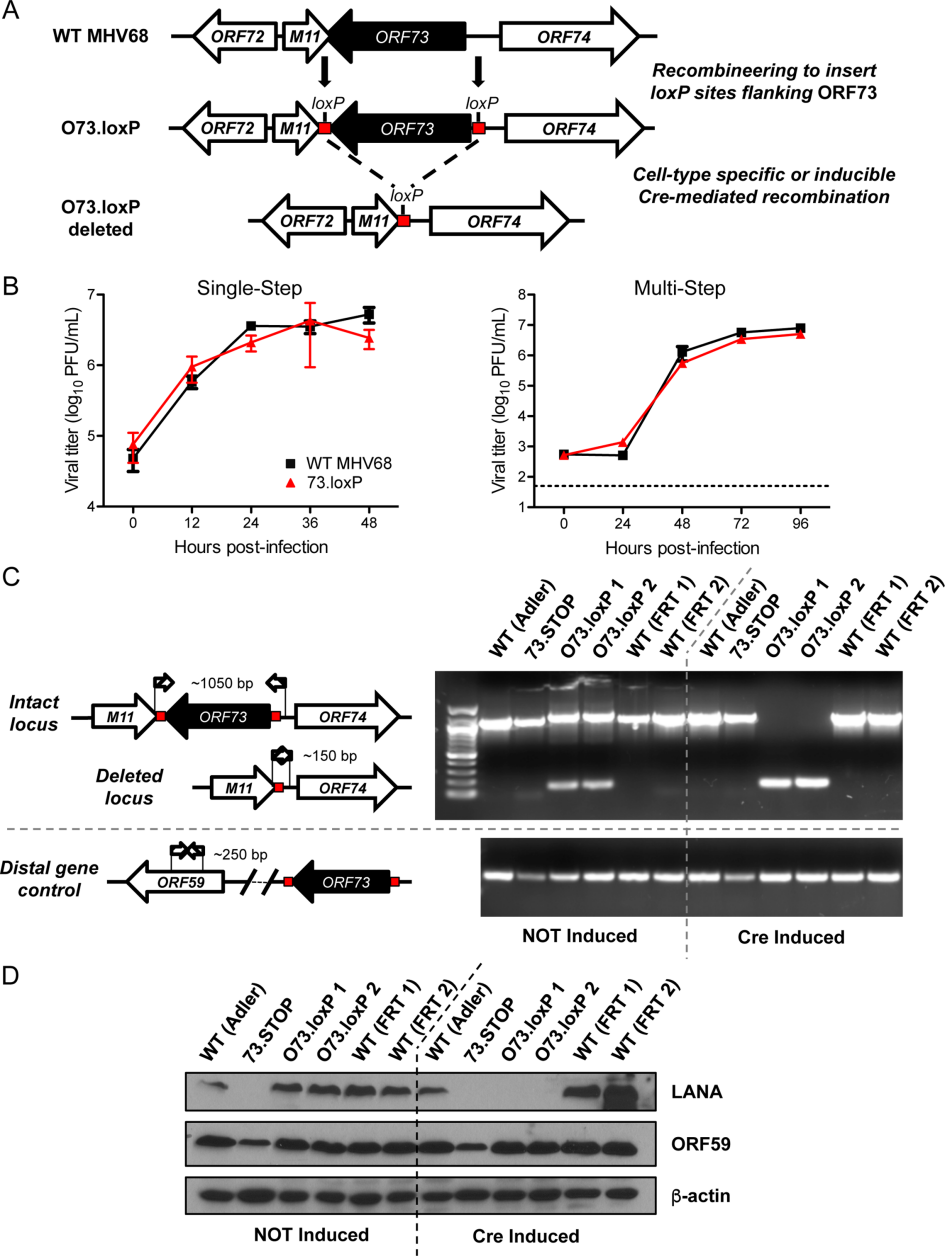
Derivation and validation of O73.loxP MHV68. (A) Schematic depicting the insertion of *loxP* sites flanking *ORF73* in the MHV68 genome and its deletion in the presence of Cre recombinase. (B) 3T3 fibroblasts were infected with WT MHV68 or O73.loxP at an MOI of 5 PFU/cell (single-step, left panel) or 0.05 PFU/cell (multi-step, right panel). Viral titers were determined by plaque assay at the indicated times post-infection. Results are means of triplicate samples. Error bars represent standard deviations. (C and D) 3T3 fibroblasts that encode Cre-ERT2 were treated with vehicle (NOT induced) or 4-hydroxytamoxifen (Cre induced) to induce Cre activity 24 h prior to infection. Treated cells were infected with the indicated viruses at an MOI of 0.05 PFU/cell. (C) Total DNA was isolated on day 4 post-infection, and PCR was performed as illustrated in the schematic to detect the intact or deleted *ORF73* locus or the distal *ORF59* locus as a control. (D) Cells were lysed on day 4 post-infection, and proteins were resolved by SDS-PAGE. Immunoblot analyses were preformed using antibodies to detect the indicated proteins. Cellular β-actin serves as a loading control.

To enable conditional deletion of the mLANA-encoding *ORF73* gene, we made an MHV68 recombinant in which *loxP* sites were inserted flanking *ORF73* (O73.loxP MHV68, **Fig 1A**). For comparative purposes, we also re-derived the previously characterized mLANA-null MHV68 [73.STOP, [19]] in the newly generated FRT BAC. O73.loxP MHV68 replicated equivalently to WT MHV68 in tissue culture in both low- and high-MOI growth analyses (**Fig 1B**). To verify that the *loxP*-flanked (floxed) *ORF73* gene could be deleted from O73.loxP MHV68, we infected Cre-ERT2 3T3 fibroblasts, cells that constitutively express Cre recombinase fused to a modified estrogen receptor [26], with WT MHV68, 73.STOP, or O73.loxP and then evaluated several MHV68 genomic loci by PCR. Though the *ORF73* locus was predominately intact in O73.loxP-infected cells treated with vehicle, only the *ORF73* deletion product was detected following activation of Cre by treatment of the cells with 4-hydroxytamoxifen (4-OHT) (**Fig 1C**). The spurious deletion observed in untreated cells is most likely the result of “leaky” Cre activity or homologous recombination between *loxP* sites [5]. *ORF73* of WT MHV68 and 73.STOP were unaffected by Cre activity. The adjacent *ORF72* gene and the distal *ORF59* gene were examined by PCR for off-target effects and found to be unaffected in all viruses tested (**S4 Fig** and **Fig 1C**, respectively). As expected, deletion of floxed *ORF73* by Cre induction resulted in an absence of detectable *orf73* transcripts in RT-PCR analyses (**S4 Fig**) and mLANA protein in immunoblot analyses (**Fig 1D**). Importantly, mLANA was readily detected in vehicle-treated cells, demonstrating that the presence of *loxP* sites did not prevent mLANA expression. As with PCR analyses, non-target viral protein expression by O73.loxP remained unaffected by Cre induction. Importantly, transcription of the adjacent *M11* gene was not affected by *loxP* insertion at the 3’ end of *ORF73* (**S4 Fig**). These results confirm that Cre recombinase targeted to the *ORF73* locus efficiently deleted *ORF73* resulting in loss of mLANA production.

**Fig 2:**
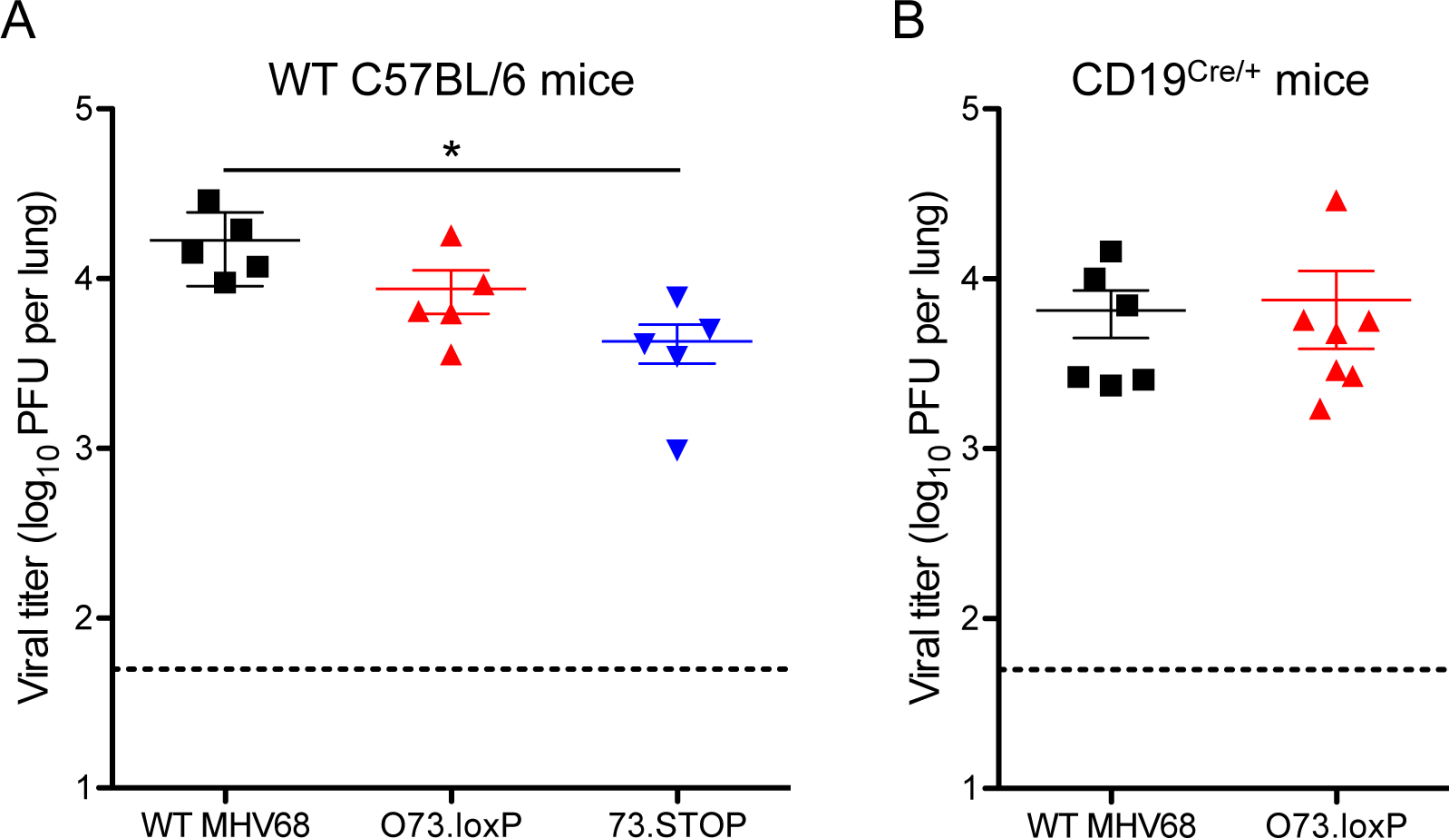
O73.loxP exhibits efficient acute replication in lungs of mice. C57BL/6 (A) or CD19^Cre/+^ (B) mice were infected IN with 1000 PFU of the indicated viruses. Mice were sacrificed on day 7 post-infection, and viral titers in lung homogenates were determined by plaque assay. Each dot represents one mouse. Error bars represent standard error of the means. * denotes p < 0.05 in a two-tailed student’s t-test.

**Fig 3:**
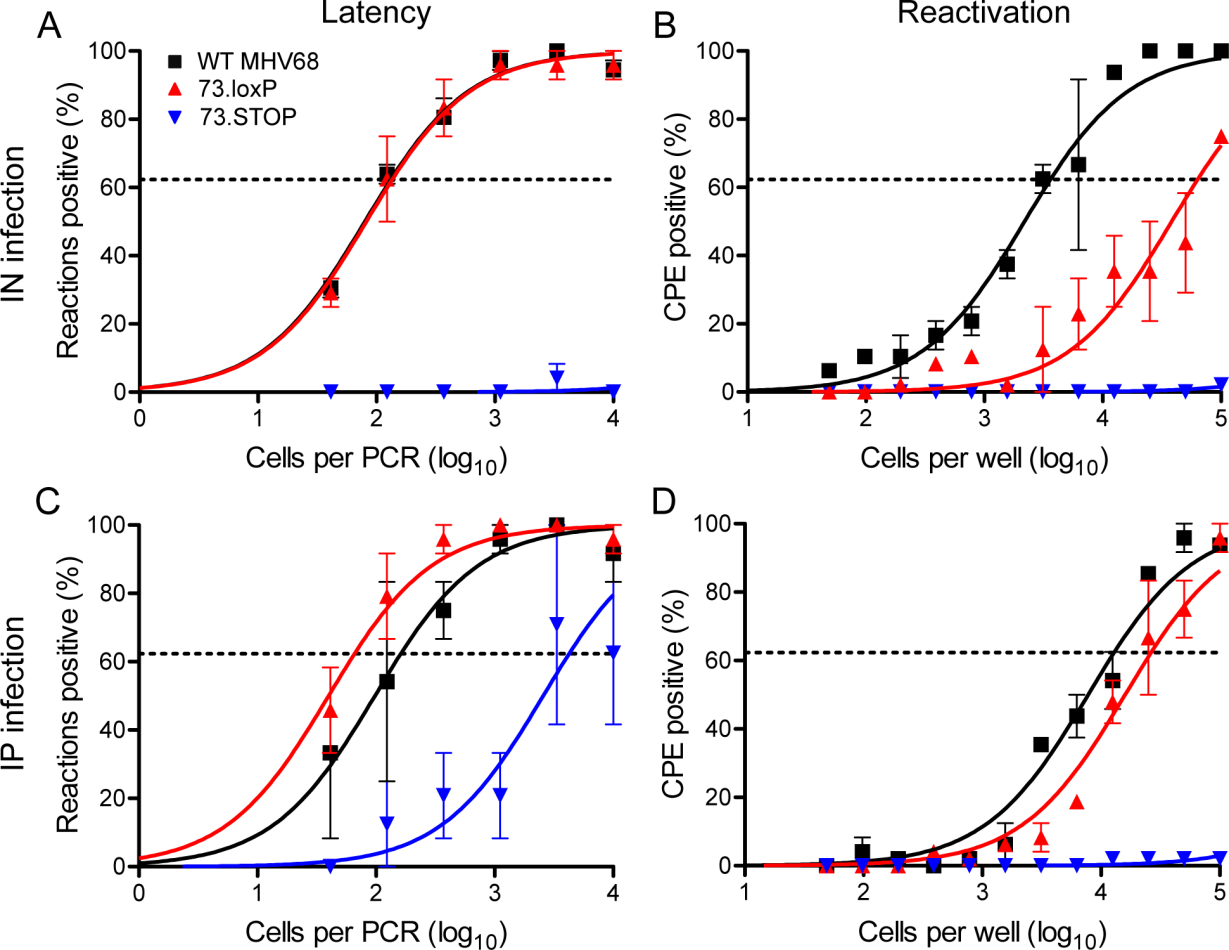
O73.loxP establishes latency in spleens of C57BL/6 mice following IN or IP inoculation. C57BL/6 mice were infected IN (A and B) or IP (C and D) with 1000 PFU of the indicated virus. Mice were sacrificed on days 16–18 post-infection. (A and C) Single-cell suspensions of spleen cells were serially diluted, and frequencies of cells harboring MHV68 genomes were determined using a limiting-dilution PCR analysis. (B and D) Reactivation frequencies were determined by *ex vivo* plating of serially diluted cells on an indicator monolayer. Cytopathic effect was scored 2–3 weeks post-plating. Groups of 3–5 mice were pooled for each infection and analysis. Results are means of 2–3 independent infections. Error bars represent standard error of the means.

Given the critical role of mLANA in MHV68 chronic infection in mice, we tested the possibility that insertion of *loxP* sites flanking *ORF73* would subtly disrupt mLANA function and attenuate O73.loxP *in vivo*. To test for such a possibility, we evaluated mice at various stages of infection following inoculation with O73.loxP compared to WT MHV68 and mLANA-null 73.STOP control viruses. With respect to acute replication, seven days after IN inoculation of WT C57BL/6 mice mean titers of WT and O73.loxP MHV68 in lungs were similar (**Fig 2A**), while 73.STOP titers were modestly attenuated relative to WT MHV68, which is consistent with previous studies [17,19]. Latency establishment by O73.loxP also was equivalent to WT MHV68 16–18 days after IN inoculation of WT mice; as expected, 73.STOP was minimally detected (**Fig 3A, Table 1**). Equivalent frequencies of WT MHV68 and O73.loxP genome-positive cells were detected in splenocytes following IP inoculation (**Fig 3C**), which is a more permissive route of infection that presumably allows the virus direct access to target cells for latency in the spleen. 73.STOP was detected at a ca. 50-fold reduced level (**Fig 3C**), consistent with previous observations [20,21]. The efficiency of O73.loxP reactivation from spleens, measured using an explant cytopathic effect assay [27], was impaired relative to WT virus following IN inoculation of WT mice (**Fig 3B, Table 2**), but a defect was not observed following IP infection (**Fig 3D**). As expected, O73.STOP was undetected in both assays. Since mLANA is necessary for latency establishment [18,19], these findings indicate that mLANA function is not impaired by the presence of *loxP* sites flanking *ORF73* in the MHV68 genome. Because mLANA also is necessary for MHV68 reactivation from the spleen following IP inoculation [20,21], these data also demonstrate that O73.loxP is not generally impaired for reactivation. However, the partial impairment of O73.loxP in reactivation from the spleen following IN inoculation suggests the possibility that homologous recombination between *loxP* sites may have occurred during latency establishment, or perhaps *ex vivo* during reactivation [5]. Together these findings indicate that the presence of *loxP* sites flanking *ORF73* in O73.loxP does not impair MHV68 latency establishment or reactivation from the spleen following direct IP inoculation; however, reactivation of O73.loxP from the spleen following IN inoculation should be considered in light of a possible defect.

**Table 1:** Frequency of viral-genome positive cells.

Mouse Strain	Virus	Route of infection	Cell population	Day post-infection^*a*^	Frequency of latent infection^*b*^
**C57BL/6**	WT MHV68 (Adler)	i.n.	Splenocyte	16	1/80
	WT MHV68 (FRT)	i.n.	Splenocyte	16	1/140
	73.loxP	i.n.	Splenocyte	16	1/140
	73.STOP	i.n.	Splenocyte	16	BLD^c^
	WT MHV68 (Adler)	i.p.	Splenocyte	16	1/170
	WT MHV68 (FRT)	i.p.	Splenocyte	16	1/160
	73.loxP	i.p.	Splenocyte	16	1/70
	73.STOP	i.p.	Splenocyte	16	1/4500*
**CD19**^**Cre/+**^	WT MHV68	i.n.	Splenocyte	16	1/190
	73.loxP	i.n.	Splenocyte	16	BLD*
	WT MHV68	i.p.	Splenocyte	16	1/70
	73.loxP	i.p.	Splenocyte	16	1/660*
	WT MHV68	i.p.	PEC	16	1/80
	73.loxP	i.p.	PEC	16	1/200
	WT MHV68	i.n.	MLN	10	1/170
	73.loxP	i.n.	MLN	10	1/2100*
	73.STOP	i.n.	MLN	10	1/1900*
	WT MHV68	i.n.	MLN	16	1/200
	73.loxP	i.n.	MLN	16	1/800*
	73.STOP	i.n.	MLN	16	BLD*
	WT MHV68	i.n.	Blood	16	1/2600
	73.loxP	i.n.	Blood	16	BLD*
	73.STOP	i.n.	Blood	16	BLD*
**AID**^**Cre/+**^	WT MHV68	i.n.	Splenocyte	16	1/100
	73.loxP	i.n.	Splenocyte	16	BLD*
	73.STOP	i.n.	Splenocyte	16	BLD*

^*a*^ Mice were sacrificed on day 10 or days 16–18 post infection.

^*b*^ Frequencies determined by Poisson distribution.

^*c*^ Below limit of detection of 1 positive PCR in 10,000 total cells as determined by Poisson distribution.

* Denotes p < 0.05 compared to WT MHV68 in two-way ANOVA with Bonferroni correction.

**Table 2:** Frequency of reactivation-competent cells.

Mouse Strain	Virus	Route of infection	Cell population	Day post-infection^*a*^	Reactivation frequency^*b*^
**C57BL/6**	WT MHV68 (Adler)	i.n.	Splenocyte	16	1/5600
	WT MHV68 (FRT)	i.n.	Splenocyte	16	1/3900
	73.loxP	i.n.	Splenocyte	16	1/65000*
	73.STOP	i.n.	Splenocyte	16	BLD^c^*
	WT MHV68 (Adler)	i.p.	Splenocyte	16	1/19000
	WT MHV68 (FRT)	i.p.	Splenocyte	16	1/14000
	73.loxP	i.p.	Splenocyte	16	1/27000
	73.STOP	i.p.	Splenocyte	16	BLD*
**CD19**^**Cre/+**^	WT MHV68	i.n.	Splenocyte	16	1/12000
	73.loxP	i.n.	Splenocyte	16	BLD*
	WT MHV68	i.p.	Splenocyte	16	1/12000
	73.loxP	i.p.	Splenocyte	16	BLD*
	WT MHV68	i.p.	PEC	16	1/1900
	73.loxP	i.p.	PEC	16	1/900

^*a*^ Mice were sacrificed on days 16–18 post infection.

^*b*^ Frequencies determined by Poisson distribution.

^*c*^ Below limit of detection of 1 positive PCR in 100,000 total cells as determined by Poisson distribution.

* Denotes p < 0.05 compared to WT MHV68 in two-way ANOVA with Bonferroni correction.

### LANA functions in CD19^+^ cells to facilitate MHV68 latency establishment

LANA-null 73.STOP MHV68 is attenuated in acute replication in the lungs and fails to establish latency in spleens of mice after IN inoculation [18,19]. RT-PCR analyses and experiments that employed an MHV68 recombinant virus encoding an mLANA-beta lactamase fusion protein to “mark” mLANA-expressing cells indicate that mLANA is expressed in B cells during MHV68 latency establishment and maintenance [14,15]. However, whether mLANA functions in B cells to facilitate MHV68 lytic replication or latency establishment in the spleen after IN inoculation is not known.

**Fig 4:**
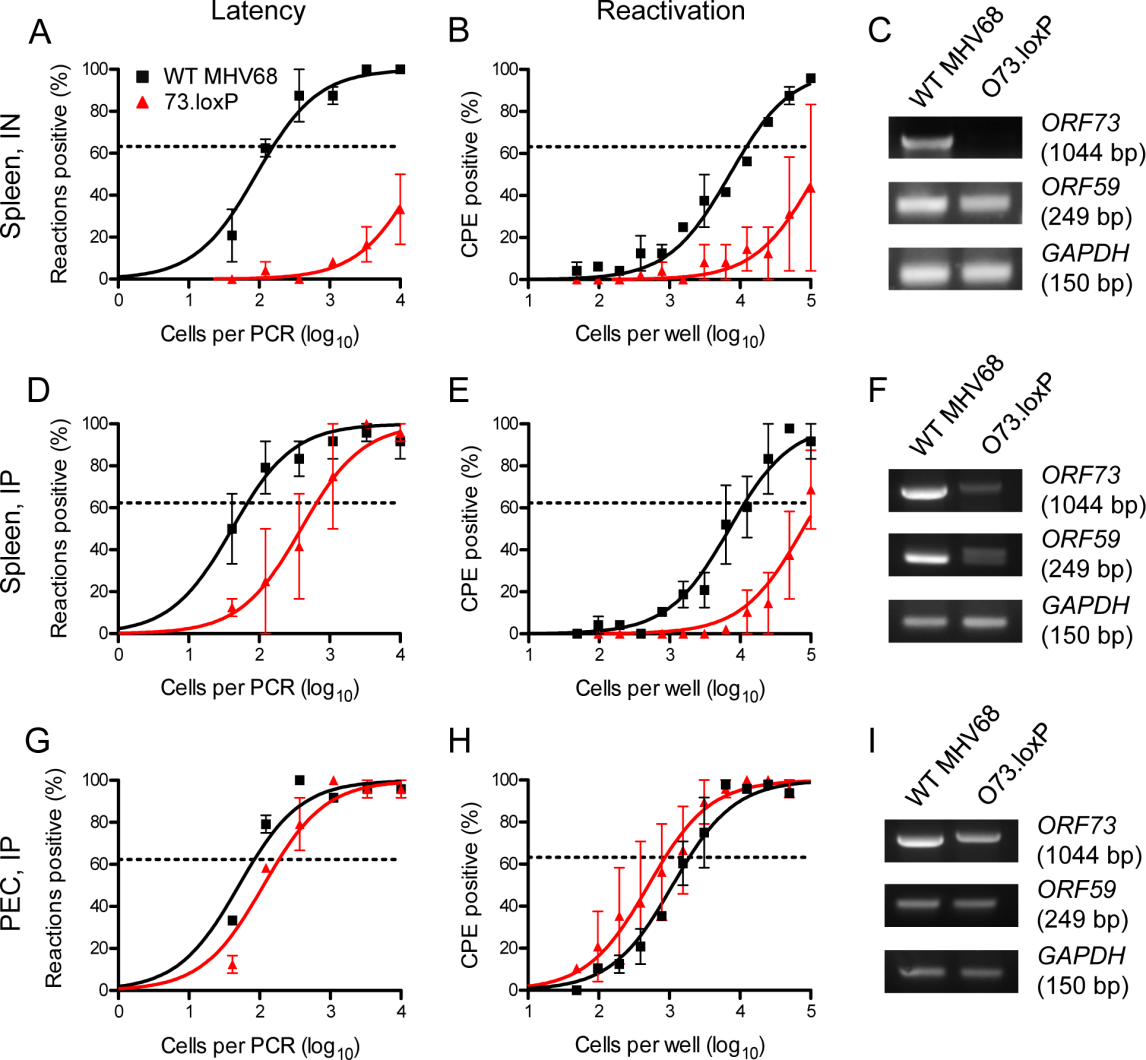
Infection of CD19^Cre/+^ mice with O73.loxP results in *ORF73* deletion and impaired splenic latency and reactivation. CD19^Cre/+^ mice were infected IN (A-C) or IP (D-I) with 1000 PFU of the indicated virus. Mice were sacrificed on days 16–18 post-infection. (A, D, and G) Single-cell suspensions of splenocytes (A and D) or PECs (G) were serially diluted, and frequencies of cells harboring MHV68 genomes were determined using a limiting-dilution PCR analysis. (B, E and H) Reactivation frequencies were determined by *ex vivo* plating of serially-diluted cells on an indicator monolayer. Cytopathic effect was scored 2–3 weeks post-plating. Groups of 3–5 mice were pooled for each infection and analysis. Results are means of 2–3 independent infections. Error bars represent standard error of the means. (C, F, and I) Total DNA was isolated from spleens (C and F) or PECs (I) at the time of harvest. PCR was performed as illustrated in Fig 1 to detect the indicated viral loci or cellular *GAPDH* as a control.

To define the relationship between mLANA expression in B cells and acute and latent infection, we evaluated O73.loxP and WT MHV68 infection in mice that express Cre recombinase under the control of the B cell-specific CD19 promoter [CD19^Cre/+^ [28]] allowing deletion of floxed *ORF73* in B cells. Seven days after IN inoculation of CD19^Cre/+^ mice, titers of O73.loxP and WT MHV68 were equivalent in lungs (**Fig 2B**). In contrast, while WT MHV68 established latency in CD19^Cre/+^ spleen at a frequency similar to that observed in WT mice (1 in 150 cells viral-genome positive), O73.loxP latency establishment was severely attenuated in the spleen following IN inoculation, with frequencies below the limit of detection of 1 in 10,000 cells (**Fig 4A**). This approximates the latency establishment defect of mLANA-null 73.STOP after IN infection of WT mice (see Fig 3A). As expected, given the results in Fig 4A, O73.loxP reactivation was severely impaired and below the limit of detection of 1 in 100,000 cells (**Fig 4B**). PCR analyses of the *ORF73* and *ORF59* loci performed on DNA isolated from spleens of infected CD19^Cre/+^ mice demonstrated that floxed *ORF73* was deleted *in vivo*, while *ORF59* remained intact (**Fig 4C**). These results indicate that functional deletion *in vivo* of *ORF73* in B cells is critical for latency establishment in the spleen following IN inoculation. Furthermore, the absence of a phenotype during acute infection suggests that mLANA expression in B cells is not a major contributor to acute viral replication in the lung.

After IP inoculation of CD19^Cre/+^ mice, O73.loxP MHV68 exhibited reduced latency establishment as measured by viral genome-positive cells in the spleen compared to WT virus, but no defect was observed in peritoneal exudate cells (PECs, **Fig 4D and 4G**). Accordingly, in a non-quantitative analysis of viral gene deletion, detection of *ORF73* by PCR was reduced in DNA isolated from spleens, but not PECs (**Fig 4F and 4I**). O73.loxP reactivation was reduced for splenocytes in a manner that correlated directly with latent viral loads, but was not diminished for PECs (**Fig 4E and 4H**). These data indicate that deletion of *ORF73* from CD19^+^ cells reduces MHV68 latency in the spleen, even after IP inoculation, but has no effect on latency in PECs. The latter interpretation is consistent with macrophages, and not B cells, serving as the primary latency reservoir for MHV68 in the peritoneal cavity [8]. Together, these results demonstrate that mLANA function in B cells plays a critical role in MHV68 latency establishment, especially following IN inoculation.

### mLANA facilitates hematogenous dissemination of MHV68 via B cells

**Fig 5:**
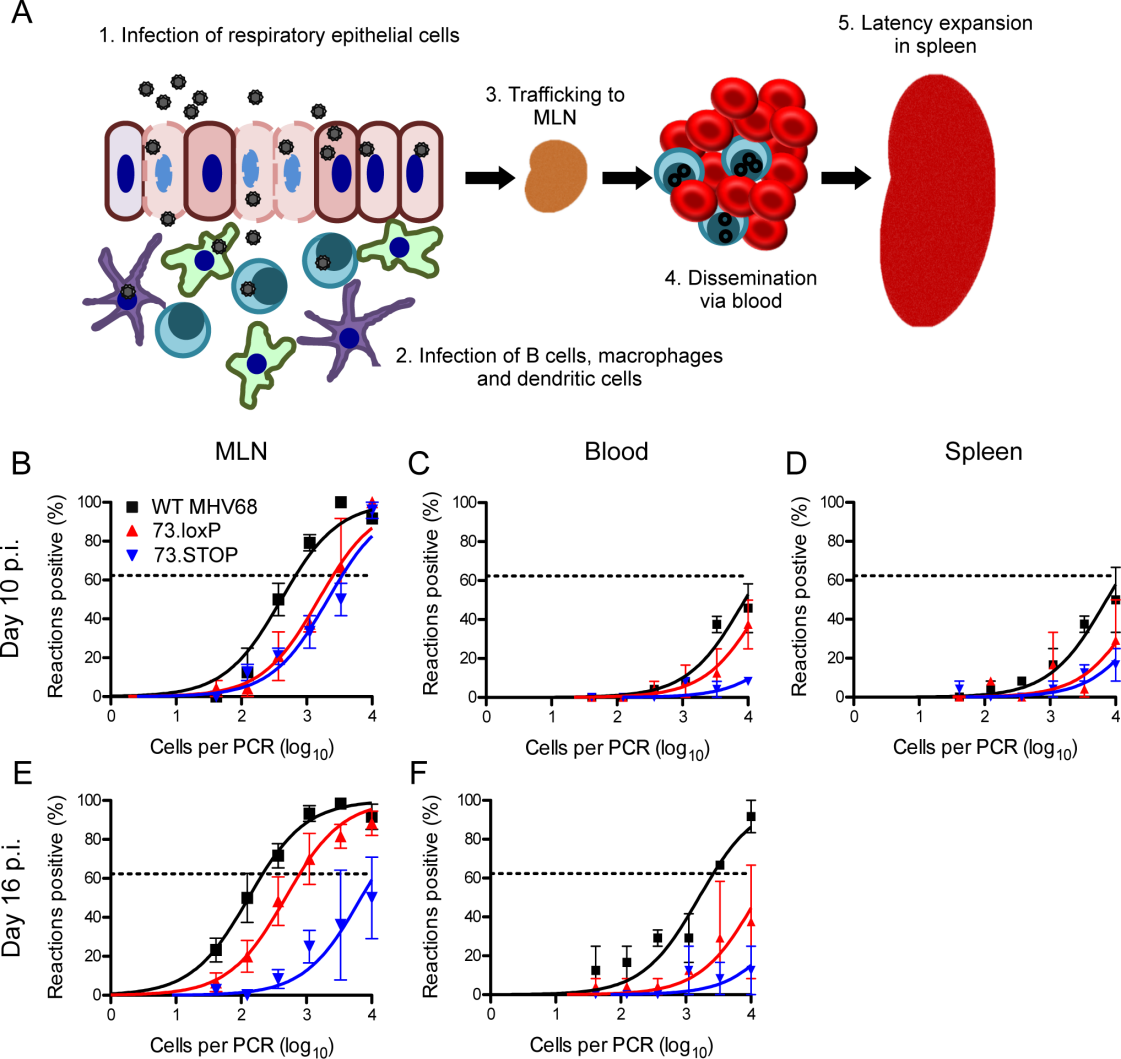
B cells control MHV68 blood-borne dissemination to the spleen in an mLANA-dependent manner. (A) A working model depicting the steps by which MHV68 is thought to traffic to the spleen following IN inoculation of mice. (B-F) CD19^Cre/+^ mice were infected IN with 1000 PFU of the indicated virus. Mice were sacrificed on day 10 (B-D) or 16 (E and F) post-infection, and MLNs (B and E), blood leukocytes, (C and F) or splenocytes (D) were isolated. Single-cell suspensions were serially diluted, and frequencies of cells harboring MHV68 genomes were determined using a limiting-dilution PCR analysis. Groups of 3–5 mice were pooled for each infection and analysis. Results are means of 2–5 independent infections. Error bars represent standard error of the means.

MHV68 disseminates systemically via lymphatic and hematogenous routes after IN inoculation. Viral transit occurs through the stepwise infection of various cell types, including epithelial, myeloid, and lymphoid cells, at different anatomical sites [5,6,29–31]. A working model of MHV68 systemic dissemination is shown in **Fig 5A**. Studies in MuMT mice suggest that B cells are necessary for systemic dissemination of MHV68 after IN infection [7,9], but this has not been directly tested. Given the cell-type-specific loss-of-function phenotype exhibited by O73.loxP in CD19^Cre/+^ mice, we reasoned that these infections could be harnessed to better define B cell roles and restriction points in the process of MHV68 dissemination following IN inoculation.

We therefore evaluated O73.loxP, 73.STOP, and WT MHV68 in kinetic analyses of infection in draining LNs (mediastinal LNs, MLN), blood leukocytes, and spleens following IN inoculation of CD19^Cre/+^ mice. In MLNs, all three viruses were detected on day 10 post-infection, with O73.loxP and 73.STOP present at approximately 5-fold lower frequencies than WT MHV68 (**Fig 5B**). By day 16 post-infection, the frequencies of cells harboring WT MHV68 or O73.loxP increased to similar levels (1 in 200 and 1 in 800, respectively). mLANA-null 73.STOP was slightly diminished compared to day 10, remaining close to the limit of detection of 1 infected cell in 10,000 total cells (**Fig 5E**). A minimal amount of preformed infectious virus indicative of ongoing lytic viral replication was present in MLNs of mice day 10 post-infection (**S5 Fig**). It is possible that lytic replication occurring in the MLN contributes to seeding infection in this organ, but it is unlikely that this minimal amount of preformed infectious virus influenced frequency determinations. PCR analyses performed on DNA isolated from infected MLNs demonstrated that *ORF73* was deleted in MLNs (**S6 Fig**). Though mLANA is not an absolute requirement for transit from lungs to spleens in severely immunocompromised mice lacking the type I interferon receptor [20], the failure of 73.STOP to expand in MLNs demonstrates the importance of mLANA in initial steps of dissemination. By extension, the minimal reduction in MLN latency observed for O73.loxP relative to WT virus, suggests that B cells are not critically involved in viral deposition in the MLN and initial expansion. Alternatively, it is possible that B cells mediate initial latency expansion in MLNs in a manner that is not dependent on mLANA.

In the blood, the number of genome positive cells was generally low for all three viruses on day 10 post-infection (**Fig 5C**). Extrapolated frequencies of genome-positive cells of 1 in 16,000 and 1 in 41,000 were determined for WT virus and O73.loxP, respectively, while 73.STOP was not definable. Though the frequency of cells latently-infected with WT MHV68 increased to ca. 1 in 2800 by day 16 post-infection, latency in the blood for O73.loxP and 73.STOP was negligible (< 1 in 100,000 genome-positive cells; **Fig 5F**). Likewise, though WT MHV68 was present in the spleen at an extrapolated frequency of ca. 1 in 15,000 cells on day 10 post-inoculation, O73.loxP and 73.STOP were minimally detected (< 1 in 100,000 and ca. 1 in 81,000 cells, respectively). Since O73.loxP established latency in MLNs but failed to efficiently spread through the blood, these data support a model in which B cells that express mLANA serve as a critical conduit for viral hematogenous dissemination and invasion of the spleen. By extension, these data indicate that MHV68 passage through B cells represents a critical bottleneck to hematogenous viral dissemination.

### mLANA is required in AID-expressing splenocytes for MHV68 latency

**Fig 6:**
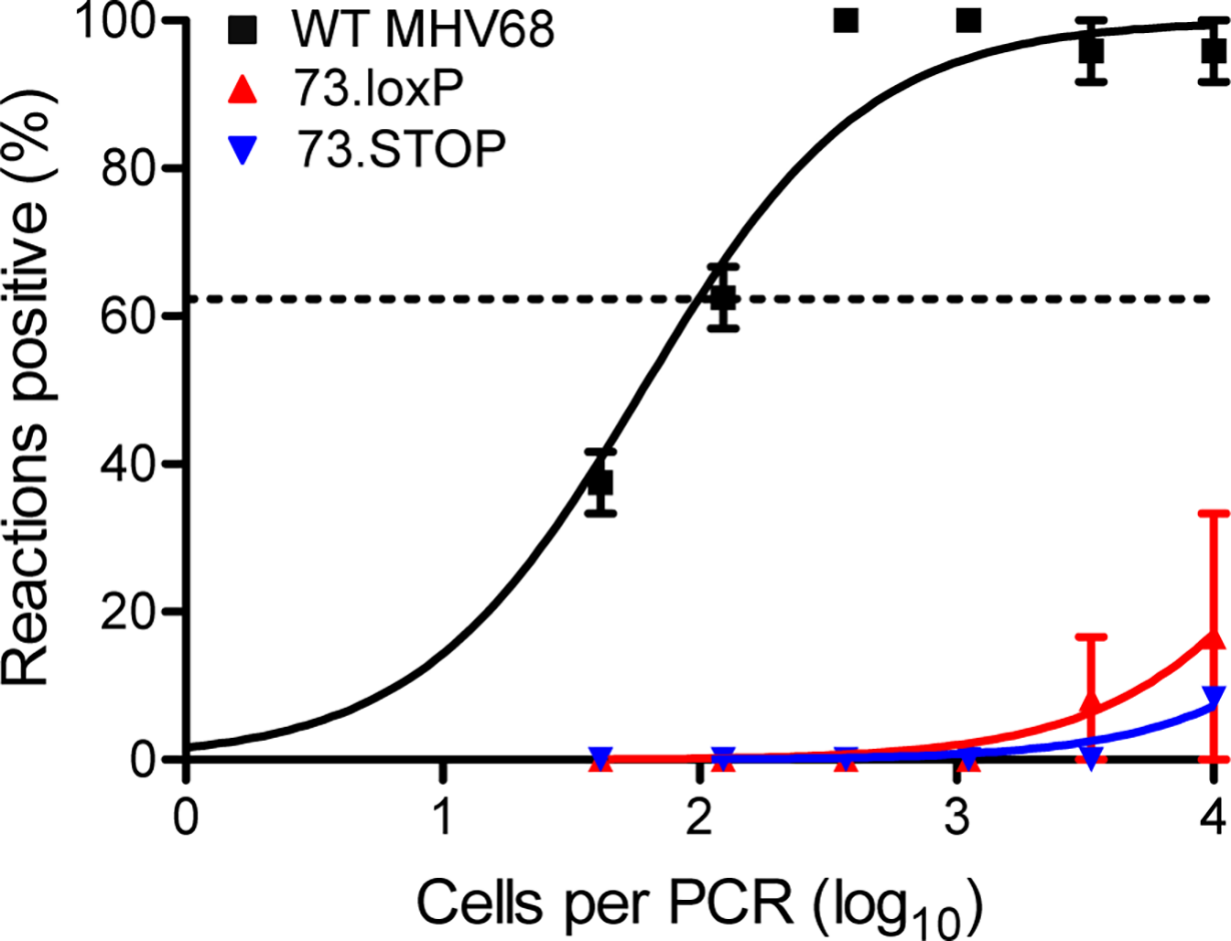
O73.loxP latency in the spleen is severely impaired following infection of AID^Cre/+^ mice. AID^Cre/+^ mice were infected IN with 1000 PFU of the indicated virus. Mice were sacrificed on days 16–18 post-infection. Single-cell suspensions of splenocytes were serially diluted, and frequencies of cells harboring MHV68 genomes were determined using a limiting-dilution PCR analysis. Results are means of 2 independent infections. Error bars represent standard error of the means.

MHV68 infects and exploits germinal center (GC) B cells to expand the number of latently-infected cells in the spleen [32–35]. In addition, a high percentage of mLANA-expressing splenocytes are GC B cells [15], and mLANA stabilizes c-myc, which may facilitate GC responses in infected cells [36]. LANA homologs facilitate maintenance of the viral episome during cell division, which appears to be a fundamental requirement for establishing and maintaining viral latency during the rapid cellular proliferation that characterizes GC reactions. As a direct test of the need for mLANA in GC reactions as a mechanism to establish latency, we evaluated O73.loxP latency after IN inoculation of AID^cre/+^ mice, which encode Cre under control of the activation-induced cytidine deaminase (AID) gene promoter [37]. AID is expressed in GC B cells to mediate class switch recombination and somatic hypermutation in the immunoglobulin gene locus [38,39]. While WT MHV68 established latency normally, neither O73.loxP nor mLANA-null 73.STOP were detected in the spleens of AID^Cre/+^ mice on day 16 post-infection (**Fig 6**). These data indicate that mLANA is necessary in AID-expressing GC B cells for latency establishment in the spleen.

### Selective deletion of mLANA after latency establishment impairs maintenance of splenic latency

**Fig 7:**
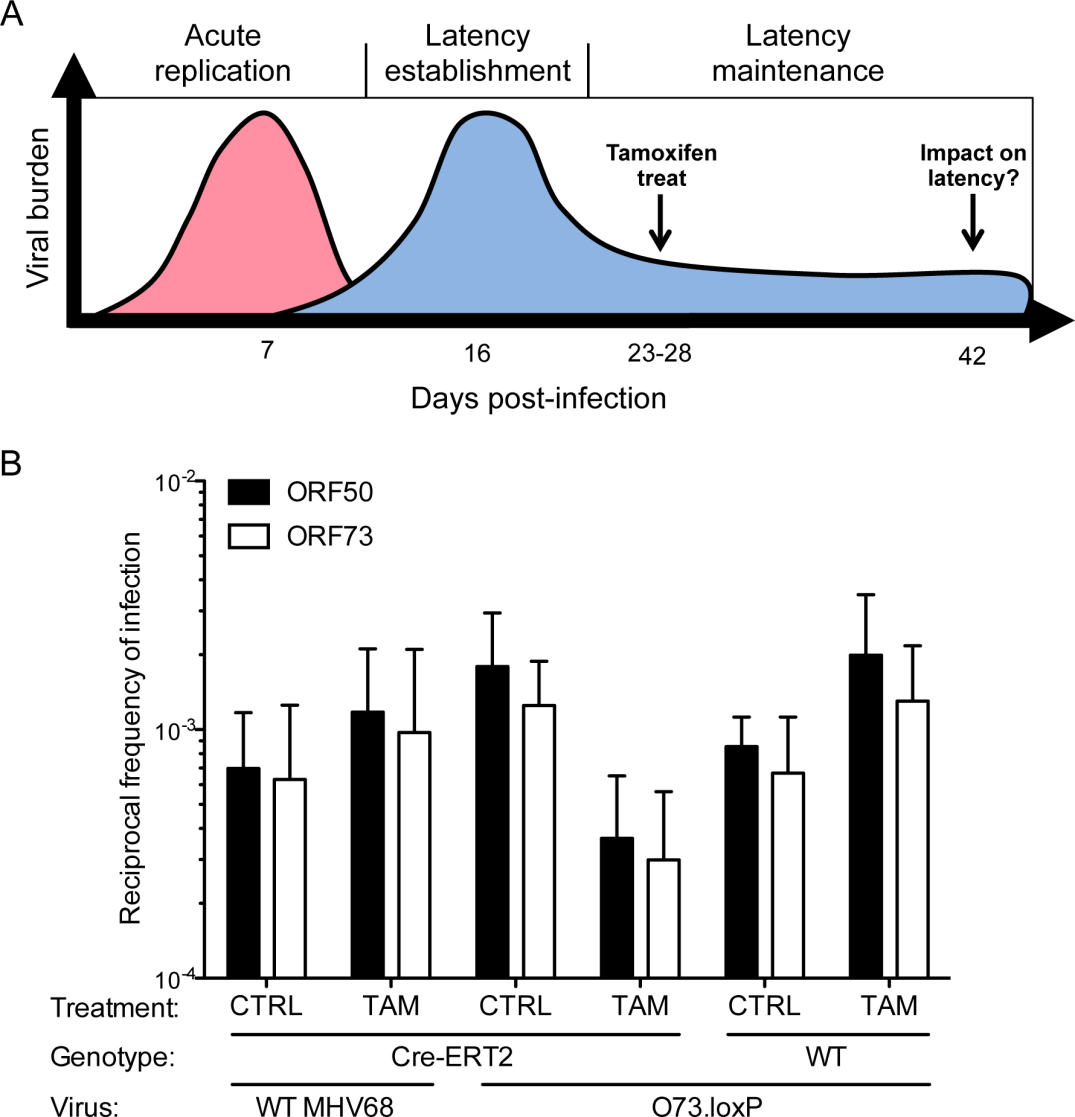
Cre-mediated deletion of *ORF73* yields a reduction in previously established MHV68 latency. (A) Schematic depicting the working model and experimental strategy for selective Cre-mediated deletion of ORF73 during MHV68 latency maintenance. (B) WT or Hemizygous Cre-ERT2 transgenic mice were infected IN with 1000 PFU of the indicated virus. On days 23–28 post-infection, mice were injected IP with vehicle or tamoxifen (2 mg/dose; one dose per day for 5 consecutive days) to induce Cre-ERT2 nuclear translocation. Mice were sacrificed on day 42 post-infection. Single-cell suspensions of splenocytes were serially diluted, and frequencies of cells harboring MHV68 genomes were determined using a limiting-dilution PCR analysis to detect either *ORF50* or *ORF73* genomic loci. Note: *ORF73* primer pairs overlap the deletion junction. Therefore, the target sequence is lost upon Cre-mediated deletion of *ORF73*. Results are means of 2 independent infections. Error bars represent standard error of the means. CTRL = mice treated with corn oil; TAM = mice treated with tamoxifen.

Given that mLANA must be present in B cells for latency establishment following IN inoculation (see Fig 3) and that MHV68 genomes fail to form circular episomes after IP inoculation with 73.STOP [20], it has not been possible to separate potentially distinct roles for mLANA in latency establishment and long-term maintenance of the viral genome *in vivo*. To test the hypothesis that mLANA is required for latency maintenance *in vivo*, we intranasally inoculated Cre-ERT2 transgenic mice, which encode a tamoxifen-inducible Cre gene in all tissues [26], with O73.loxP. WT MHV68 and WT mice that do not encode Cre-ERT2 were infected as controls and to detect potential effects on latency resulting from tamoxifen treatment. Beginning on day 23 post-infection, after latency had been established in the spleen, tamoxifen was administered for five consecutive days to induce Cre activity and the resultant deletion of floxed *ORF73*. The schematic in **Fig 7A** outlines the timeline followed for this experiment. Two weeks after completing tamoxifen treatments (day 42 post-infection), spleens were harvested and the frequencies of latently infected cells were quantified by LD-PCR. For these analyses we used two different primer sets: one that amplifies *ORF50*, which should not be affected by *ORF73* deletion, and another that spans the 5’ end of *ORF73*, the 5’ loxP site, and adjacent sequence upstream of *ORF73*. This amplicon should be absent upon *ORF73* deletion, enabling a direct evaluation of cells that retain viral genomes (*ORF50*^+^) despite loss of mLANA-encoding *ORF73*. Validation experiments in cultured cells confirmed that *ORF50*- and *ORF73*-specific primers were equally sensitive for detecting viral genomes and that *ORF73* primers did not amplify, while *ORF50* primers did amplify, a product upon *ORF73* deletion by Cre (**S7 Fig**).

In both Cre-ERT2 mice infected with WT MHV68 and WT mice infected with O73.loxP, tamoxifen treatment resulted in a slight increase in genome-positive cells compared to mice treated with vehicle control (**Fig 7B, Table 3**). Genome frequencies were nearly identical as measured by *ORF50* and *ORF73* primer sets. However, relative to vehicle control-treated mice, tamoxifen treatment resulted in an 8-fold decrease in the number of splenocytes harboring MHV68 genomes in Cre-ERT2 mice infected with O73.loxP (**Fig 7B**). Notably, a similar reduction was observed using both *ORF50-* and *ORF73*-directed primer sets. These findings indicate that Cre-mediated deletion of *ORF73* following establishment of latency promoted a loss of MHV68 genomes from latently infected spleens and demonstrate that mLANA is critical for maintenance of long-term MHV68 latency.

**Table 3:** Reciprocal frequencies of ORF50^+^ and ORF73^+^ cells for vehicle or tamoxifen-treated Cre-ERT2 or WT mice infected with O73.loxP or FRT MHV68.

Virus & Genotype^a^	Vehicle			Tamoxifen			VEH/ TAM^e^
	*ORF50*^b^	*ORF73*^c^	*ORF50/ORF73*^d^	*ORF50*	*ORF73*	*ORF50/ORF73*	
O73.loxP/Cre-ERT2	410	610	1.5	2000	2300	1.2	4.70*
O73.loxP/WT	1300	2400	1.9	360	550	1.5	0.27
WT/Cre-ERT2	970	930	0.96	540	560	1.0	0.56

^a^ Virus = WT or O73.loxP MHV68; Genotype = mouse genotype (Cre-ERT2 or WT littermate).

^b^ Reciprocal frequency of *ORF50*+ cells (1 in ‘x’ cells).

^c^ Reciprocal frequency of *ORF73*+ cells (1 in ‘x’ cells).

^d^ Ratio of frequency of *ORF50*+ cells to frequency of *ORF73*+ cells. A ratio near 1 demonstrates that genomes were detected at similar frequencies with both primer pairs.

^e^ Ratio of *ORF50*+ cells from vehicle-treated mice to *ORF50*+ cells from tamoxifen-treated mice. A ratio near 1 indicates no effect of treatment. A number greater than 1 indicates a relative reduction in viral genomes.

* Denotes p < 0.05 compared to WT MHV68 in two-tailed unpaired student’s t-test.

## Discussion

In this study, we describe the generation and characterization of O73.loxP, a recombinant MHV68 that permits the Cre-dependent, conditional deletion of *ORF73 in vivo*. This system differs from traditional viral mutagenesis approaches in that it allows for cell-type specific and inducible deletion of a gene of interest rather than complete ablation. Since mLANA is a multifunctional viral protein that facilitates both lytic replication and latency establishment, we utilized this new reagent to better define mLANA’s functions in specific cell types and at certain stages of viral infection.

### Experimental considerations

There are potential caveats to Cre-lox approaches to control the timing of viral gene deletion. Insertion of *loxP* sites within a viral genome may inadvertently impact transcription efficiency, impact splicing, or directly alter the function of overlapping noncoding RNAs. With regard to O73.loxP, the *loxP* sites flanking *ORF73* did not overtly influence viral replication or latency establishment in mice lacking Cre. Given the potent attenuation of mLANA-null and other *ORF73*-mutant viruses [19–21], this shows that the *loxP* insertions did not disrupt mLANA expression. Although viral reactivation efficiency after IN inoculation was reduced for O73.loxP, reactivation occurred normally after IP inoculation. Given the necessity for mLANA in reactivation after IP infection [20,21], this indicates that mLANA in O73.loxP is capable of normal function during reactivation. In light of findings by Stevenson and colleagues indicating that homologous recombination between *loxP* sites within the MHV68 genome is possible [5], we reason that a similar phenomenon may be at work in our experiments following IN inoculation and after viral dissemination to the spleen.

Although it appears from our data that Cre-mediated excision of floxed viral genes is very efficient for cell-type-specific Cre expression, it remains possible that not every Cre-encoding cell will delete the targeted locus. And, while viral genomes were reduced in mice following tamoxifen induction of Cre, a proportion of cells infected with O73.loxP did not delete *ORF73*. Whether this represents an issue specifically in B cells, difficulty in targeting viral genomes that are potentially epigenetically modified, or an intrinsic inefficiency in the inducible Cre mice is not yet clear. Nonetheless, the dramatic phenotypes observed in our studies demonstrate the utility of this approach to better define the necessity of specific viral genes in a time- and tissue-specific fashion in a small animal model of GHV pathogenesis.

### Functions of mLANA in latency establishment and maintenance

Previous studies using traditional mutagenesis approaches demonstrated that both the absence of mLANA and point mutations in the DNA binding domain of mLANA lead to a profound defect in latency establishment in the spleen after IN inoculation [18,19,21]. However, these mutations also result in defects in lytic viral replication [17,19,21,22]. Since lytic replication in the lung is important for MHV68 dissemination to the spleen after IN inoculation [40], it remained possible that defects in latency establishment observed with mLANA-null or mutant virus were indirect, the result of upstream defects in acute replication and dissemination. This idea was further supported by the observation that mLANA-null and mutant viruses establish and maintain splenic latency after IP inoculation [20,21]. The finding that viral titers in the lungs of CD19^Cre/+^ mice infected with WT or O73.loxP MHV68 were equivalent, yet O73.loxP latency in the same mice was strongly attenuated, indicates that defective lytic replication in the lung in the absence of *ORF73* is not the sole explanation for defective latency observed with 73.STOP.

Provided *ORF73* deletion occurs rapidly upon B cell infection, our results also suggest that B cells, which are infected by MHV68 in mouse lungs [40], are not major contributors to productive viral replication in the lungs. Consistent with these observations, a study utilizing a recombinant MHV68 that encodes a switchable, floxed fluorescent marker confirmed that B cell-derived virus was undetectable in the lungs during the first 7–10 d post IN infection of CD19^Cre/+^ mice [41]. It therefore is likely that mLANA functions in mucosal epithelial cells of the lung to promote efficient lytic replication.

Although it is clear from our studies that mLANA expression in B cells is necessary for dissemination from the lung (or MLN) to the spleen, it remains plausible that lytic replication and/or reactivation from B cells facilitates systemic infection. A direct test of this hypothesis is possible using an extension of the genetic platform described above. Conditional deletion of *ORF50* or *ORF57* in a virus in which *ORF73* is intact, with resultant defects in the establishment of latency in the spleen following IN infection of CD19^Cre/+^ mice, would provide strong evidence that lytic replication in B cells is necessary for systemic MHV68 infection.

Based on studies from the Stevenson laboratory demonstrating that floxed fluorescent loci in the MHV68 genome were recombined in mice expressing Cre in myeloid cells, indicating passage of MHV68 through that compartment, our working model depicts MHV68 trafficking from epithelial barriers to draining lymphoid organs via dendritic cells and/or macrophages [5,31]. Reduced latent infection of MLNs by mLANA-null virus, but only minimal impact on O73.loxP infection of CD19^Cre/+^ mice, suggests the possibility that mLANA functions in non-B cells to permit efficient trafficking to the lung, or is necessary for seeding and expansion of B cells in the MLN after trafficking. Our data are consistent with *ORF73* deletion not occurring until B cell expansion is underway in MLNs, or indicate that mLANA is not required for latency amplification in MLN B cells once a virus encoding mLANA is deposited in the lymph node via a non-B cell route. The latter point seems less likely given the importance of mLANA in maintaining viral genomes during cell division.

Since O73.loxP expanded in MLNs after IN inoculation of CD19^Cre/+^ mice, but failed to attain WT levels in blood, we propose that mLANA functions in B cells to permit efficient hematogenous dissemination and viral colonization of the spleen. A failure of O73.loxP MHV68-infected B cells to proliferate or a gradual loss or dilution of viral genomes resulting from a lack of mLANA-mediated episome maintenance during B cell expansion are possible mechanisms. Failed latency establishment by O73.loxP in AID^Cre/+^ mice demonstrates the requirement for mLANA in GC B cells. Moreover, mLANA stabilizes c-myc to promote cellular proliferation [36], and, although not evaluated in B cells *per se*, ectopically expressed mLANA induces promoters for genes that drive cell cycle progression [36,42]. Use of the H2b-YFP MHV68 recombinant [33] coupled with floxed *ORF73* could directly evaluate proliferation and gene expression in peripheral B cells upon *ORF73* deletion.

Maintenance of the viral episome is perhaps the most extensively studied function of the KSHV LANA protein [10,43], and mLANA also supports the replication of plasmids containing a terminal repeat *in vitro* [24]. However, whether episome maintenance, primarily defined in cell culture, is required for maintenance of latency *in vivo* is unknown. The KSHV genome contains an autonomous replication element that is LANA-independent [44], and shRNA-mediated depletion of kLANA does not “cure” PEL cell lines of KSHV [45]. Likewise, LANA-null and LANA DNA-binding mutant MHV68 are maintained long-term in splenic B cells after IP inoculation, despite a modest defect in early establishment [20,21]. However, the severe latency establishment defect observed following infection of AID^cre/+^ mice is most consistent with a model in which mLANA facilitates viral genome maintenance in rapidly proliferating GC B cells. Moreover, the reduction in O73.loxP infected splenocytes following induction of Cre activity *after* latency establishment supports the long-standing hypothesis that mLANA is necessary for genome maintenance during long-term chronic infection, although it is not yet clear whether infection is primarily depleted in the subset of GC B cells that remain infected long-term or in memory B cells [15,32–35].

### Further defining mechanisms of systemic infection

MHV68 spreads from epithelial barriers to draining lymph nodes via myeloid cells [5,29,31]. However, cell types that mediate viral trafficking to the spleen and systemic infection are less clear. The observation that CD19^+^ B cells constitute a much greater proportion of MHV68-infected cells in MLNs than do CD11c+ and CD11b+ myeloid cells (~100 to 1000 fold higher) suggests that B cell expansion in the LN precedes hematogenous spread [6]. This is in concordance with the necessity for B cells in splenic latency establishment after IN inoculation of MuMT mice mentioned above [7,9]. The deficit in O73.loxP latency in blood-borne leukocytes of CD19^Cre/+^ mice adds new support to the hypothesis that B cells are the key vehicles for transport of MHV68 to the spleen. Once in the spleen, it is likely that MHV68 usurps GC reactions in order to facilitate latency establishment. Indeed, GC reactions supported by T follicular helper (T_FH_) cells and IL-21 are critical for the expansion of MHV68 latency in the spleen [46,47]. The latency defect exhibited by O73.loxP MHV68 in AID^cre/+^ mice emphasizes the importance of MHV68 passage through GC B cells in order to successfully colonize the host. Together, the defects in latency establishment by O73.loxP in CD19^Cre/+^ and AID^Cre/+^ mice add a viral-genetic component to models suggesting that MHV68 makes use of B cells for blood-borne dissemination and that infection of GC B cells is necessary for latency in the spleen.

## Materials and methods

### Ethics statement

Mouse experiments performed for this study were carried out in accordance with National Institutes of Health, United States Dept. of Agriculture, and UAMS Division of Laboratory Animal Medicine and Institutional Animal Care and Use Committee (IACUC) guidelines. The protocol supporting this study was approved by the UAMS IACUC (animal use protocol 3587). Mice were anesthetized prior to inoculations and sacrificed humanely at the end of experiments.

### Cells and viruses

NIH 3T12 (ATCC CCL-164) and Swiss albino 3T3 fibroblasts (ATCC CCL-92) were cultured in Dulbecco’s Modified Eagle Medium (DMEM) supplemented with 10% fetal bovine serum (FBS), 100 U/ml penicillin, 100 μg/ml streptomycin, and 2 mM L-glutamine (cMEM). Cells were cultured at 37°C with 5% CO_2_ and ~99% humidity. Murine embryonic fibroblasts (MEFs) were harvested from C57BL/6 mouse embryos and immortalized as previously described [27]. 3T3 fibroblasts encoding tamoxifen-inducible Cre recombinase were a gift from Dr. Eric J. Brown. Vero-Cre cells were originally obtained from the laboratory of Dr. David Leib. To generate 3T12 cells stably expressing Flp recombinase, BOSC23 (ATCC CRL-11270) cells first were transfected with pMSCV-Flp, a retroviral plasmid encoding Flp recombinase. Retrovirus containing supernatants were harvested at 24 and 48 h post-transfection, filtered through a 0.45 μm filter (Millipore), and used to transduce 3T12 cells. Transduced cells were selected with puromycin and expanded. Previously described viruses used in this study include BAC-derived wildtype MHV68 [25] and mLANA-null MHV68 (73.STOP) [19]. Derivation of a new WT MHV68 BAC that contains *frt* sites flanking BAC vector sequences, O73.loxP, and 73.STOP in the FRT BAC is described below. Viruses derived from MHV68 with *frt* sites flanking the BAC cassette were passaged in Flp-expressing 3T12 fibroblasts to remove the BAC cassette.

### Mouse infections and tissue harvests

Male and female C57BL/6, CD19^Cre/+^ (B6.129P2(C)-Cd19tm1(cre)Cgn/J), AID ^Cre/+^ (B6.129P2-Aicdatm1(cre)Mnz/J), and inducible Cre-ERT2 (B6.Cg-Tg(UBC-cre/ERT2)1Ejb/J) mice were purchased from Jackson Laboratories. Mice were bred and maintained according to all local, state, and federal guidelines under the supervision of the Division of Laboratory Animal Medicine at the University of Arkansas for Medical Sciences. Eight-to-ten week old mice were anesthetized using isoflurane and inoculated with 1000 PFU of virus diluted in incomplete DMEM (20 μl) for IN inoculations or injected with 1000 PFU of virus diluted in incomplete DMEM (100 μl) for IP inoculations. Splenocytes and peritoneal exudate cells (PECs) were harvested as described previously [20]. Cells from mediastinal lymph nodes (MLNs) were harvested by homogenizing pooled MLNs on cell strainers and resuspending in DMEM. Blood was extracted by cardiac puncture and deposited into conical tubes pre-coated with EDTA and containing 200 μl of heparin sulfate solution (1000 U/ml). Buffy coats were prepared using Lympholyte-M solution for cell separation (Cedarlane #cI5030).

### *In vivo* tamoxifen treatment

Tamoxifen treatment of Cre-ERT2 transgenic mice was performed as described previously [26,48]. Briefly, 8 to 10 week old, male and female Cre-ERT2 mice or their wild-type littermates were infected intranasally with 1000 PFU of either FRT or O73.loxP MHV68. Twenty-three days after infection, mice were injected IP with either 2 mg of tamoxifen (Sigma Aldrich #T5648) dissolved in 98% corn oil and 2% ethanol or vehicle control once a day for 5 consecutive days.

### Limiting-dilution analyses

Limiting-dilution (LD) analyses to quantify the frequency of latently infected cells from spleen, MLNs, blood or peritoneum were performed as described previously [8]. Briefly, cells from infected mice were plated in three-fold serial dilutions for a total of 6 dilutions (12 wells per dilution) in a background of 10^4^ uninfected 3T12 fibroblasts per well. Cells were digested by proteinase K treatment at 56°C overnight. Cell extracts were then subjected to two rounds of nested PCR using gene-specific primers to either an *ORF50* target [8] or to *ORF73* (73USoutF and 73USoutR as the outer primer pair and 73USnestF and 73nestR as the nested primer pair; see **S1 Table**). PCR products were resolved in a 1.5% agarose gel.

PCR for the detection of the full-length *ORF73* gene was performed utilizing primers 73_IG_DS and 73_IG_US (**S1 Table**) docking at genomic coordinates 103881–103907 and 104900–104924, respectively, proximal to ORF73 using Taq polymerase (Sydlabs) and reaction conditions: 94°C for 2 min; 94°C for 30 sec, 55°C sec, 72°C for 1 min 30 sec for 45 cycles; 72°C for 10 min; 4°C for indefinite time. *ORF59* and *GAPDH* genes were detected by PCR utilizing primers 59PCR1 and 59PCR2 (**S1 Table**) for *ORF59* and GAPDHF and GAPDHR for *GAPDH*, as described previously [49].

To determine the frequency of reactivating cells [27], splenocytes or PECs harvested from infected mice were resuspended in cMEM (10^6^ cells/ml) and were plated in two-fold serial dilutions on 96 well tissue culture plates containing an indicator monolayer of MEFs. Separate samples of mechanically disrupted cells also were plated on MEF monolayers to detect preformed infectious virus. Cell monolayers were evaluated for the presence of CPE 14 and 21 days post-plating.

### Immunoblot analyses

Immunoblot analyses were performed as previously described [49]. Briefly, cells were lysed with radio immunoprecipitation (RIPA) buffer (150 mM NaCl, 20 mM Tris, 2 mM EDTA, 1% NP-40, 0.25% deoxycholate supplemented with phosphatase and protease inhibitors), and protein samples were centrifuged at 16,000 xg to remove insoluble debris. Protein content for each sample was quantified using the BioRad DC Protein Assay (BioRad). Samples were diluted in 2X Laemmli sample buffer and resolved by sodium dodecyl sulfate polyacrylamide gel electrophoresis (SDS-PAGE) and transferred to nitrocellulose membranes (Thermo Scientific). Blots were probed with the indicated primary antibodies and with horseradish peroxidase (HRP) conjugated secondary antibodies (Jackson ImmunoResearch). Chemiluminescent signal was detected using a ChemiDoc MP Imaging System (Bio-Rad) on blots treated with SuperSignal Pico West ECL reagent (Thermo Scientific) or Clarity ECL reagent (Bio-Rad).

### Fluorescence microscopy

Live cells were imaged by fluorescence microscopy at the indicated time points using 20X magnification on an Eclipse Ti-U fluorescent microscope (Nikon). Images were acquired with a D5-QilMc digital camera and analyzed using NIS-Elements software (Nikon).

### Plaque assays

Plaque assays were performed as previously described [49]. Briefly, infected cells were lysed by two freeze-thaw cycles. Ten-fold serial dilutions of lysates were added to monolayers of NIH 3T12 cells (2 x 10^5^ cells/well) that were plated the previous day. For determining viral titers from infected organs, lungs were homogenized by mechanical disruption (5 cycles) in a Mini-Beadbeater-16 (Biospec Products), and homogenates were freeze-thawed twice and serially diluted (10-fold) in cMEM. For all plaque assays, plates were rocked every 15 min for 1 h at 37°C. Infected cells were overlaid with 1.5% methylcellulose in cMEM supplemented with 5% FBS and incubated at 37°C for 7–8 days. Methylcellulose media was then aspirated, and cell monolayers were stained with a solution of crystal violet (0.1%) in formalin to facilitate the identification and quantification of plaques.

### Generation of recombinant viruses

The WT MHV68 BAC was created by *en passant* mutagenesis [50]. To replace the *loxP* sites with *frt* sites on the original WT MHV68 BAC [25], primers containing a 40 bp homologous BAC cassette sequence adjacent to one of the *loxP* sites, the 34 bp *frt* site sequence, and a sequence homologous to a kanamycin (Kan) selection cassette (KanFRT1_Fwd and KanFRT1_Rev, **S1 Table**) were used to amplify the kanamycin selection cassette and *Isce*I recognition site from the plasmid pEPKanS2 by PCR [50]. The resulting amplicon was digested with *Dpn*I to remove template DNA, excised and purified from an agarose gel, and electroporated into a competent E. coli strain GS1783.5 harboring the original WT MHV68 BAC. After recovery, the electroporated cells were plated on Luria broth (LB) agar plates containing chloramphenicol (30 μg/ml) and kanamycin (25 μg/ml) and allowed to grow at 30°C for 48 h. Transformants were screened by colony PCR using primers specific to the Kan resistance cassette. Positive colonies were inoculated into LB broth containing chloramphenicol and 2% arabinose to induce *Isce*I expression and excision of the Kan marker, followed by substitution of the target *loxP* site for a *frt* site by Red-mediated recombination. Recombined clones were screened for the presence of *frt* by PCR and verified by sequencing. The second *frt* site was introduced in a second round of *en passant* mutagenesis utilizing primers KanFRT2_Fwd and KanFRT2_Rev (**S1 Table**) for amplification of the Kan cassette from pEPKanS2 by PCR.

To generate the O73.loxP BAC, *loxP* sites were inserted adjacent to the 5’ and 3’ ends of *ORF73* in a FRT BAC template by two successive rounds of *en passant* mutagenesis utilizing primers 73loxpR1_fwd and 73loxpR1_rev for the first round and primers 73loxpR2_fwd and 73loxpR2_rev (**S1 Table**) for the second round of mutagenesis, following the procedure outlined for the generation of the FRT BAC. The 3’ end of *M11* was not disrupted by insertion of *loxP* sites. The 3’ end of *M11* was regenerated when inserting the *loxP* site at the 3’ end of *ORF73* in a manner that maintained the natural coding and transcription termination sequence of *M11*. Similar approaches were previously used to maintain M11 sequence when manipulating the 3’ end of *ORF73* [15,51]. 73.STOP FRT was generated by introducing a premature stop codon and frameshift mutation into *ORF73* on a FRT BAC template by *en passant* mutagenesis utilizing primers 73stopFRT_fwd and 73stopFRT_rev (**S1 Table**). Viruses were passaged in Flp-expressing 3T12 fibroblasts to remove the BAC cassette, and titers were quantified as described previously [49].

### Antibodies and drug treatments

Antibodies used in this study include goat polyclonal anti-GFP (Rockland Immunochemicals, Inc, #600-101-215), rabbit polyclonal mLANA anti-serum [21], mouse polyclonal MHV68 anti-serum [49], chicken anti-ORF59 IgY (Gallus Immunotech), and mouse monoclonal anti-β-actin (Sigma Aldrich, #A2228). Fluorophore-conjugated secondary antibodies used in this study include AlexaFluor donkey anti-goat 488, AlexaFluor goat anti-mouse 568, and AlexaFluor goat anti-chicken 568 (Life Technologies). For drug treatments, the 17β-estradiol agonist Z-4-hydroxytamoxifen (4-OHT; Alexis Biochemicals; #ALX-550-361-M001) was dissolved at a stock concentration of 2 mM. Inducible-Cre 3T3 fibroblasts plated the previous day were treated with either 0.2 μM 4-OHT to induce nuclear translocation of Cre or ethanol as a vehicle control for 24 h prior to infection.

### Plasmids and transfections

pMSCV-Flp was generated by first amplifying the Flp recombinase ORF from pCMV14-Flp utilizing Flp-specific primers that also encode overhangs for restriction enzymes *Bam*HI and *Eco*RI (forward primer FlpR_BglII and reverse primer FlpR_EcoRI, **S1 Table**). The resulting amplicon was digested with *Bam*HI and *Eco*RI along with pMSCV. Both the PCR product and pMSCV were resolved by gel electrophoresis, gel-purified, and ligated using T4 DNA ligase (New England Biolabs; #M0202). Competent DH5α *E*. *coli* were transformed with the ligated product, and after recovery, a small inoculum was plated on LB ampicillin plates. Positive clones were screened by restriction digest and sequenced to ensure proper insertion of the Flp ORF into pMSCV. All transfections were performed using Lipofectamine and Plus Reagent (Life Technologies) according to the manufacturer’s instructions.

### Statistics

All statistical analyses were performed using GraphPad Prism software (GraphPad Software, San Diego, CA). Statistical significance was determined using two-way ANOVA with Bonferroni correction or by a two-tailed unpaired Student's t test with a 95% confidence.

## Supporting information

S1 Table List of primers used in this study. (PDF)

S1 Fig Modification of the MHV68 BAC to enable Cre-mediated deletion of viral genes. (A) Schematic depicting the strategy for modifying the MHV68 BAC by first replacing the *loxP* sites that flanked the BAC cassette with *frt* sites, followed by insertion of *loxP* sites that flanked *ORF73*. (B) *En passant* mutagenesis was performed on the parental MHV68 BAC (lane 1) to generate a new WT MHV68 BAC (FRT BAC, lanes 2 and 3). A subsequent round of *en passant* mutagenesis was performed on the FRT BAC to generate O73.loxP (lane 4) and 73.STOP (lane 5) BACs. BAC DNA was digested with the indicated restriction endonucleases, and digestion products were resolved by agarose-gel electrophoresis to evaluate the gross genetic integrity of the newly derived BACs. The mutation in 73.STOP generates a new XbaI site that results in a ~8 kb digestion product (see lane 5) not present in the other BACs. (C) The parental Adler (lane 1) and FRT (lane 2) BACs were digested with the indicated restriction endonucleases, and digestion products were resolved by gel eclectrophoresis. Although a larger than expected band is present for Adler BAC digested with *Eco*RI, the double digest that includes *Not*I, an enzyme that cuts once in each copy of the terminal repeat, results in an identical banding pattern for both BACs. This indicates that the large DNA fragments produced by single digests of the Adler BAC are due to the presence of more copies of the terminal repeats relative to FRT BAC constructs.
(TIF)

S2 Fig Flp recombinase mediates excision of the FRT-flanked BAC cassette. (A) Schematic depicting Flp-dependent removal of the BAC cassette, which encodes GFP, from the MHV68 genome. (B) 3T12 fibroblasts were stably transduced with empty vector or Flp recombinase encoding retroviruses and used to generate MHV68 stocks. For visualization and confirmation of BAC removal, cells were infected with WT MHV68 or O73.loxP grown in either Flp- or Flp+ cells at an MOI of 0.05 PFU/cell. Plaques were analyzed on day 4 post-infection by brightfield and fluorescence microscopy for the presence or absence of GFP.
(TIF)

S3 Fig FRT BAC-derived MHV68 exhibits normal replication, latency establishment, and reactivation. (A and B) 3T3 fibroblasts were infected with either Adler BAC- or FRT BAC-derived WT MHV68 at an MOI of 5 PFU/cell (single-step growth curve, A) or 0.05 PFU/cell (multi-step growth curve, B). Viral titers were determined at the indicated times post-infection by plaque assay. Results are means of triplicate samples. Error bars represent standard deviations. (C-E) C57BL/6 mice were infected IN with 1000 PFU of either Adler BAC- or FRT BAC-derived WT MHV68. (C) Mice were sacrificed on day 7 post-infection, and viral titers in lung homogenates were determined by plaque assay. Each dot represents one mouse. Error bars represent standard error of the means. (D and E) Mice were sacrificed on days 16–18 post-infection. (D) Single-cell suspensions of spleen cells were serially diluted and frequencies of cells harboring MHV68 genomes were determined using a limiting-dilution PCR analysis. (E) Reactivation frequencies were determined by *ex vivo* plating of serially diluted cells on an indicator monolayer. Cytopathic effect was scored 2–3 weeks post-plating. Groups of 3–5 mice were pooled for each infection and analysis. Results are means of three independent infections. Error bars represent standard error of the means.
(TIF)

S4 Fig Cre-mediated deletion of *ORF73* does not impact the adjacent *ORF72* or *M11* genes. (A) 3T3 fibroblasts that encode Cre-ERT2 were treated with vehicle or 4-hydroxytamoxifen (4-OHT) to induce Cre activity 24 h prior to infection. Treated cells were infected with FRT BAC-derived WT MHV68 (isolate 1, lanes 3 and 9; isolate 2, lanes 4 and 10), O73.loxP (isolate 1; lanes 5 and 11; isolate 2, lanes 6 and 12), Adler BAC-derived WT MHV68 (lanes 1 and 7), or mLANA-null 73.STOP (lanes 2 and 8) at an MOI of 0.05 PFU/cell. Total DNA was isolated on day 4 post-infection, and PCR was performed as illustrated in the schematic to detect the indicated viral loci or cellular *GAPDH* as a control. (B) 3T12 fibroblasts or Vero cells constitutively expressing Cre recombinase were infected with WT MHV68 or O73.loxP MHV68 at an MOI of 0.1 PFU/cell. RNA was isolated on day 4 post-infection, and reverse transcription reactions were performed to with and without RT to generate cDNA. PCR was performed to detect the indicated viral transcripts. Products were resolved by agarose gel electrophoresis.
(TIF)

S5 Fig Viral replication in MLNs is minimal on day 10 post-infection. CD19^Cre/+^ mice were infected IN with 1000 PFU of the indicated viruses. Mice were sacrificed on day 10 post-infection and MLNs were harvested. Single-cell suspensions were subjected to hypotonic and mechanical lysis. Lysates were plated in a limiting-dilution manner on an indicator monolayer to quantify preformed infectious virus. Cytopathic effect was scored 2–3 weeks post-plating. Groups of 3–5 mice were pooled for each infection and analysis. Results are means of two independent infections. Error bars represent standard error of the means.
(TIF)

S6 Fig *ORF73* is deleted in MLNs of CD19^Cre/+^ mice. CD19^Cre/+^ mice were infected IN with 1000 PFU of O73.loxP MHV68. Mice were sacrificed on days 10 or 16 post-infection, and total DNA was isolated from mediastinal lymph nodes. PCR was performed to detect the indicated viral or cellular genes, and products were resolved by agarose gel electrophoresis. The additional samples represent comparative controls as a means to evaluate *ORF73* deletion in the presence or absence of Cre recombinase.
(TIF)

S7 Fig Validation of *ORF73*-specific LD-PCR. 3T12 fibroblasts or Vero cells constitutively expressing Cre recombinase were infected with WT MHV68 or O73.loxP MHV68 at an MOI of 0.1 PFU/cell. Cells were harvested on day 4 post-infection. Single-cell suspensions were serially diluted, and frequencies of cells harboring MHV68 genomes were determined using a limiting-dilution PCR analysis. In one set of analyses, primers specific for *ORF50* were used. In another set of analyses, primers specific for *ORF73* were used. Non-linear regression analyses were performed to determine the frequencies of cell harboring viral genomes. Viral genomes were equivalently detected by both primer sets in 3T12 fibroblasts lacking Cre. Viral genomes were detected with *ORF50* primers, but not *ORF73* primers, when cells expressing Cre were infected. Results are means of two independent experiments. Error bars represent standard error of the means. N.D. = not definable.
(TIF)

## References

[1] Damania B (2007) DNA tumor viruses and human cancer. Trends Microbiol 15: 38–44. doi: 10.1016/j.tim.2006.11.002 17113775 10.1016/j.tim.2006.11.002

[2] Knipe DM, Howley PM (2013) Fields virology Philadelphia, PA: Wolters Kluwer/Lippincott Williams & Wilkins Health. 2 volumes p.

[3] Barton E, Mandal P, Speck SH (2010) Pathogenesis and host control of gammaherpesviruses: lessons from the mouse. Annu Rev Immunol 29: 351–397. 10.1146/annurev-immunol-072710-081639 21219186

[4] Speck SH, Ganem D (2012) Viral latency and its regulation: lessons from the gamma-herpesviruses. Cell Host Microbe 8: 100–115. 10.1016/j.chom.2010.06.014 PMC2914632 20638646

[5] Gaspar M, May JS, Sukla S, Frederico B, Gill MB, et al (2011) Murid herpesvirus-4 exploits dendritic cells to infect B cells. PLoS Pathog 7: e1002346 doi: 10.1371/journal.ppat.1002346 22102809 10.1371/journal.ppat.1002346 PMC3213091

[6] Feldman ER, Kara M, Oko LM, Grau KR, Krueger BJ, et al (2016) A Gammaherpesvirus Noncoding RNA Is Essential for Hematogenous Dissemination and Establishment of Peripheral Latency. mSphere 1. 10.1128/mSphere.00105-15 PMC4838037 27110595

[7] Usherwood EJ, Stewart JP, Robertson K, Allen DJ, Nash AA (1996) Absence of splenic latency in murine gammaherpesvirus 68-infected B cell-deficient mice. J Gen Virol 77 (Pt 11): 2819–2825. 8922476 10.1099/0022-1317-77-11-2819

[8] Weck KE, Kim SS, Virgin HI, Speck SH (1999) Macrophages are the major reservoir of latent murine gammaherpesvirus 68 in peritoneal cells. J Virol 73: 3273–3283. 10074181 10.1128/jvi.73.4.3273-3283.1999 PMC104091

[9] Stewart JP, Usherwood EJ, Ross A, Dyson H, Nash T (1998) Lung epithelial cells are a major site of murine gammaherpesvirus persistence. J Exp Med 187: 1941–1951. 9625754 10.1084/jem.187.12.1941 PMC2212355

[10] Ballestas ME, Kaye KM (2011) The latency-associated nuclear antigen, a multifunctional protein central to Kaposi's sarcoma-associated herpesvirus latency. Future Microbiol 6: 1399–1413. doi: 10.2217/fmb.11.137 22122438 10.2217/fmb.11.137 PMC3857968

[11] Virgin HWt, Latreille P, Wamsley P, Hallsworth K, Weck KE, et al (1997) Complete sequence and genomic analysis of murine gammaherpesvirus 68. J Virol 71: 5894–5904. 9223479 10.1128/jvi.71.8.5894-5904.1997 PMC191845

[12] Cheng BY, Zhi J, Santana A, Khan S, Salinas E, et al (2012) Tiled microarray identification of novel viral transcript structures and distinct transcriptional profiles during two modes of productive murine gammaherpesvirus 68 infection. J Virol 86: 4340–4357. doi: 10.1128/JVI.05892-11 22318145 10.1128/JVI.05892-11 PMC3318610

[13] Johnson LS, Willert EK, Virgin HW (2010) Redefining the genetics of murine gammaherpesvirus 68 via transcriptome-based annotation. Cell Host Microbe 7: 516–526. doi: 10.1016/j.chom.2010.05.005 20542255 10.1016/j.chom.2010.05.005 PMC2900189

[14] Marques S, Efstathiou S, Smith KG, Haury M, Simas JP (2003) Selective gene expression of latent murine gammaherpesvirus 68 in B lymphocytes. J Virol 77: 7308–7318. doi: 10.1128/JVI.77.13.7308-7318.2003 12805429 10.1128/JVI.77.13.7308-7318.2003 PMC164786

[15] Nealy MS, Coleman CB, Li H, Tibbetts SA (2010) Use of a virus-encoded enzymatic marker reveals that a stable fraction of memory B cells expresses latency-associated nuclear antigen throughout chronic gammaherpesvirus infection. J Virol 84: 7523–7534. doi: 10.1128/JVI.02572-09 20484501 10.1128/JVI.02572-09 PMC2897616

[16] Verma SC, Lan K, Robertson E (2007) Structure and function of latency-associated nuclear antigen. Curr Top Microbiol Immunol 312: 101–136. 17089795 10.1007/978-3-540-34344-8_4 PMC3142369

[17] Forrest JC, Paden CR, Allen RD 3rd, Collins J, Speck SH (2007) ORF73-null murine gammaherpesvirus 68 reveals roles for mLANA and p53 in virus replication. J Virol 81: 11957–11971. doi: 10.1128/JVI.00111-07 17699571 10.1128/JVI.00111-07 PMC2168792

[18] Fowler P, Marques S, Simas JP, Efstathiou S (2003) ORF73 of murine herpesvirus-68 is critical for the establishment and maintenance of latency. J Gen Virol 84: 3405–3416. doi: 10.1099/vir.0.19594-0 14645921 10.1099/vir.0.19594-0

[19] Moorman NJ, Willer DO, Speck SH (2003) The gammaherpesvirus 68 latency-associated nuclear antigen homolog is critical for the establishment of splenic latency. J Virol 77: 10295–10303. doi: 10.1128/JVI.77.19.10295-10303.2003 12970414 10.1128/JVI.77.19.10295-10303.2003 PMC228443

[20] Paden CR, Forrest JC, Moorman NJ, Speck SH (2010) Murine gammaherpesvirus 68 LANA is essential for virus reactivation from splenocytes but not long-term carriage of viral genome. J Virol 84: 7214–7224. doi: 10.1128/JVI.00133-10 20444892 10.1128/JVI.00133-10 PMC2898264

[21] Paden CR, Forrest JC, Tibbetts SA, Speck SH (2011) Unbiased mutagenesis of MHV68 LANA reveals a DNA-binding domain required for LANA function in vitro and in vivo. PLoS Pathog 8: e1002906. 10.1371/journal.ppat.1002906 PMC3435236 22969427

[22] Salinas E, Byrum SD, Moreland LE, Mackintosh SG, Tackett AJ, et al (2015) Identification of Viral and Host Proteins That Interact with Murine Gammaherpesvirus 68 Latency-Associated Nuclear Antigen during Lytic Replication: a Role for Hsc70 in Viral Replication. J Virol 90: 1397–1413. doi: 10.1128/JVI.02022-15 26581985 10.1128/JVI.02022-15 PMC4719591

[23] Sifford JM, Stahl JA, Salinas E, Forrest JC (2015) Murine gammaherpesvirus-68 LANA and SOX homologs counteract ATM-driven p53 activity during lytic viral replication. J Virol. 10.1128/JVI.02867-15 PMC4810692 26676792

[24] Habison AC, Beauchemin C, Simas JP, Usherwood EJ, Kaye KM (2012) Murine gammaherpesvirus 68 LANA acts on terminal repeat DNA to mediate episome persistence. J Virol 86: 11863–11876. doi: 10.1128/JVI.01656-12 22915819 10.1128/JVI.01656-12 PMC3486315

[25] Adler H, Messerle M, Wagner M, Koszinowski UH (2000) Cloning and mutagenesis of the murine gammaherpesvirus 68 genome as an infectious bacterial artificial chromosome. J Virol 74: 6964–6974. 10888635 10.1128/jvi.74.15.6964-6974.2000 PMC112213

[26] Ruzankina Y, Pinzon-Guzman C, Asare A, Ong T, Pontano L, et al (2007) Deletion of the developmentally essential gene ATR in adult mice leads to age-related phenotypes and stem cell loss. Cell Stem Cell 1: 113–126. doi: 10.1016/j.stem.2007.03.002 18371340 10.1016/j.stem.2007.03.002 PMC2920603

[27] Weck KE, Barkon ML, Yoo LI, Speck SH, Virgin HI (1996) Mature B cells are required for acute splenic infection, but not for establishment of latency, by murine gammaherpesvirus 68. J Virol 70: 6775–6780. 8794315 10.1128/jvi.70.10.6775-6780.1996 PMC190721

[28] Rickert RC, Roes J, Rajewsky K (1997) B lymphocyte-specific, Cre-mediated mutagenesis in mice. Nucleic Acids Res 25: 1317–1318. 9092650 10.1093/nar/25.6.1317 PMC146582

[29] Frederico B, Chao B, Lawler C, May JS, Stevenson PG (2015) Subcapsular sinus macrophages limit acute gammaherpesvirus dissemination. J Gen Virol 96: 2314–2327. doi: 10.1099/vir.0.000140 25872742 10.1099/vir.0.000140 PMC4681069

[30] Frederico B, Chao B, May JS, Belz GT, Stevenson PG (2014) A murid gamma-herpesviruses exploits normal splenic immune communication routes for systemic spread. Cell Host Microbe 15: 457–470. doi: 10.1016/j.chom.2014.03.010 24721574 10.1016/j.chom.2014.03.010

[31] Frederico B, Milho R, May JS, Gillet L, Stevenson PG (2012) Myeloid infection links epithelial and B cell tropisms of Murid Herpesvirus-4. PLoS Pathog 8: e1002935 doi: 10.1371/journal.ppat.1002935 23028329 10.1371/journal.ppat.1002935 PMC3447751

[32] Collins CM, Boss JM, Speck SH (2009) Identification of infected B-cell populations by using a recombinant murine gammaherpesvirus 68 expressing a fluorescent protein. J Virol 83: 6484–6493. doi: 10.1128/JVI.00297-09 19386718 10.1128/JVI.00297-09 PMC2698576

[33] Collins CM, Speck SH (2012) Tracking murine gammaherpesvirus 68 infection of germinal center B cells in vivo. PLoS One 7: e33230 doi: 10.1371/journal.pone.0033230 22427999 10.1371/journal.pone.0033230 PMC3302828

[34] Flano E, Kim IJ, Woodland DL, Blackman MA (2002) Gamma-herpesvirus latency is preferentially maintained in splenic germinal center and memory B cells. J Exp Med 196: 1363–1372. doi: 10.1084/jem.20020890 12438427 10.1084/jem.20020890 PMC2193987

[35] Willer DO, Speck SH (2003) Long-term latent murine Gammaherpesvirus 68 infection is preferentially found within the surface immunoglobulin D-negative subset of splenic B cells in vivo. J Virol 77: 8310–8321. doi: 10.1128/JVI.77.15.8310-8321.2003 12857900 10.1128/JVI.77.15.8310-8321.2003 PMC165249

[36] Rodrigues L, Popov N, Kaye KM, Simas JP (2013) Stabilization of Myc through heterotypic poly-ubiquitination by mLANA is critical for gamma-herpesvirus lymphoproliferation. PLoS Pathog 9: e1003554 doi: 10.1371/journal.ppat.1003554 23950719 10.1371/journal.ppat.1003554 PMC3738482

[37] Robbiani DF, Bothmer A, Callen E, Reina-San-Martin B, Dorsett Y, et al (2008) AID is required for the chromosomal breaks in c-myc that lead to c-myc/IgH translocations. Cell 135: 1028–1038. doi: 10.1016/j.cell.2008.09.062 19070574 10.1016/j.cell.2008.09.062 PMC2713603

[38] Muramatsu M, Kinoshita K, Fagarasan S, Yamada S, Shinkai Y, et al (2000) Class switch recombination and hypermutation require activation-induced cytidine deaminase (AID), a potential RNA editing enzyme. Cell 102: 553–563. 11007474 10.1016/s0092-8674(00)00078-7

[39] Muramatsu M, Sankaranand VS, Anant S, Sugai M, Kinoshita K, et al (1999) Specific expression of activation-induced cytidine deaminase (AID), a novel member of the RNA-editing deaminase family in germinal center B cells. J Biol Chem 274: 18470–18476. 10373455 10.1074/jbc.274.26.18470

[40] Moser JM, Farrell ML, Krug LT, Upton JW, Speck SH (2006) A gammaherpesvirus 68 gene 50 null mutant establishes long-term latency in the lung but fails to vaccinate against a wild-type virus challenge. J Virol 80: 1592–1598. doi: 10.1128/JVI.80.3.1592-1598.2006 16415035 10.1128/JVI.80.3.1592-1598.2006 PMC1346930

[41] Vidy A, Sacher T, Adler H, Jordan S, Koszinowski UH, et al (2013) Systemic and local infection routes govern different cellular dissemination pathways during gammaherpesvirus infection in vivo. J Virol 87: 4596–4608. doi: 10.1128/JVI.03135-12 23408606 10.1128/JVI.03135-12 PMC3624335

[42] Ottinger M, Pliquet D, Christalla T, Frank R, Stewart JP, et al (2009) The interaction of the gammaherpesvirus 68 orf73 protein with cellular BET proteins affects the activation of cell cycle promoters. J Virol 83: 4423–4434. doi: 10.1128/JVI.02274-08 19244327 10.1128/JVI.02274-08 PMC2668493

[43] Ballestas ME, Chatis PA, Kaye KM (1999) Efficient persistence of extrachromosomal KSHV DNA mediated by latency-associated nuclear antigen. Science 284: 641–644. 10213686 10.1126/science.284.5414.641

[44] Verma SC, Lan K, Choudhuri T, Cotter MA, Robertson ES (2007) An autonomous replicating element within the KSHV genome. Cell Host Microbe 2: 106–118. doi: 10.1016/j.chom.2007.07.002 18005725 10.1016/j.chom.2007.07.002 PMC4287363

[45] Godfrey A, Anderson J, Papanastasiou A, Takeuchi Y, Boshoff C (2005) Inhibiting primary effusion lymphoma by lentiviral vectors encoding short hairpin RNA. Blood 105: 2510–2518. doi: 10.1182/blood-2004-08-3052 15572586 10.1182/blood-2004-08-3052

[46] Collins CM, Speck SH (2014) Expansion of murine gammaherpesvirus latently infected B cells requires T follicular help. PLoS Pathog 10: e1004106 doi: 10.1371/journal.ppat.1004106 24789087 10.1371/journal.ppat.1004106 PMC4006913

[47] Collins CM, Speck SH (2015) Interleukin 21 signaling in B cells is required for efficient establishment of murine gammaherpesvirus latency. PLoS Pathog 11: e1004831 doi: 10.1371/journal.ppat.1004831 25875847 10.1371/journal.ppat.1004831 PMC4395336

[48] Ballon G, Akar G, Cesarman E (2015) Systemic expression of Kaposi sarcoma herpesvirus (KSHV) Vflip in endothelial cells leads to a profound proinflammatory phenotype and myeloid lineage remodeling in vivo. PLoS Pathog 11: e1004581 doi: 10.1371/journal.ppat.1004581 25607954 10.1371/journal.ppat.1004581 PMC4301867

[49] Stahl JA, Paden CR, Chavan SS, MacLeod V, Edmondson RD, et al (2012) Amplification of JNK signaling is necessary to complete the murine gammaherpesvirus 68 lytic replication cycle. J Virol 86: 13253–13262. doi: 10.1128/JVI.01432-12 23015701 10.1128/JVI.01432-12 PMC3503053

[50] Tischer BK, Smith GA, Osterrieder N (2010) En passant mutagenesis: a two step markerless red recombination system. Methods Mol Biol 634: 421–430. doi: 10.1007/978-1-60761-652-8_30 20677001 10.1007/978-1-60761-652-8_30

[51] Gupta A, Oldenburg DG, Salinas E, White DW, Forrest JC (2017) Murine Gammaherpesvirus 68 Expressing Kaposi Sarcoma-Associated Herpesvirus Latency-Associated Nuclear Antigen (LANA) Reveals both Functional Conservation and Divergence in LANA Homologs. J Virol 91. 10.1128/JVI.00992-17 PMC5599733 28747501

