# Supplementary material for: Conditional mutagenesis in vivo reveals cell type- and infection stage-specific requirements for LANA in chronic MHV68 infection
Source: PLoS Pathog. 2018 Jan 24;14(1):e1006865. doi: 10.1371/journal.ppat.1006865 (PMC5798852; doi:10.1371/journal.ppat.1006865)
Supplement: S1 Table — (PDF) [file ppat.1006865.s001.pdf]

S1 Table. Primers used in this study

Primer Name	Sequence (5' to 3')	Purpose
73USoutF	AGGGAAGTGTTGGTGATGAAGG	ORF73 LD-PCR
73USoutR	CCCATACTTGAGGGACACCGTA	ORF73 LD-PCR
73USnestF	GACTCCTCATCACCTTGGCATC	ORF73 LD-PCR
73USnestR	TGACACACCTGCCTACAGAAT	ORF73 LD-PCR
KanFRT1_Fwd	CGCCGGTGTTAATTAAGGGCCGGCCGCATCAGCTTCCTAG GAAGTTCCTATTCTCTAGAAAGTATAGGAACTTCGGATCTCC CCGCCCAGCGTCTAGGGATAACAGGGTAATCGATTT	FRT BAC cloning
KanFRT1_Rev	TTCGAGTTCGCCAATGACAAGACGCTGGGCGGGGAGATCC GAAGTTCCTATACTTTCTAGAGAATAGGAACTTCCTAGGAAG CTGATGCGGCCGGCCAGTGTTACAACCAATTAACC	FRT BAC cloning
KanFRT2_Fwd	GTTGCCCAGGATATATTCTGGGAATGTGATTTATGTCTGAAT AACTTCGTATAGCATACATTATACGAAGTTATTTATGTCTGAG ACCCTTGTCCTAGGGATAACAGGGTAATCGATTT	FRT BAC cloning
KanFRT2_Rev	GATCTAGTCCAGGACAACAGGGACAAGGGTCTCAGACATA AATAACTTCGTATAATGTATGCTATACGAAGTTATTCAGACATA AATCACATTCCGCCAGTGTTACAACCAATTAACC	FRT BAC cloning
73loxpR1_fwd	CTGAGGTTGTGTTGCGTGTAGTCGGTGGGGATGTGGGCAT ATAACTTCGTATAGCATACATTATACGAAGTTATTATCTGAAAG AGATAAAGTATAGGGATAACAGGGTAATCGATTT	O73.loxP BAC cloning
73loxpR1_rev	TGCTAAAAGTTGTGACTGTGTACTTTATCTCTTTCAGATAATA ACTTCGTATAATGTATGCTATACGAAGTTATATGCCCACATCC CCACCGACGCCAGTGTTACAACCAATTAACC	O73.loxP BAC cloning
73loxpR2_fwd	GTTGCCCAGGATATATTCTGGGAATGTGATTTATGTCTGAAT AACTTCGTATAGCATACATTATACGAAGTTATTTATGTCTGAG ACCCTTGTCCTAGGGATAACAGGGTAATCGATTT	O73.loxP BAC cloning
73loxpR2_rev	GATCTAGTCCAGGACAACAGGGACAAGGGTCTCAGACATA AATAACTTCGTATAATGTATGCTATACGAAGTTATTCAGACATA AATCACATTCCGCCAGTGTTACAACCAATTAACC	O73.loxP BAC cloning
73stopFRT_fwd	GAGGAGGGGCTGGTCTTTTTGGAGCGCGGCGTCTTTTAGG TCTAGACGGCTGCTGGTTTGTTGAAGCTAGGGATAACAGG GTAATCGATTT	73STOP.FRT BAC cloning
73stopFRT_rev	ATCAGGGTGCAAACGTAGGTGCTTCAACAAACCAGCAGCC GTCTAGACCTAAAAGACGCCGCGCTCCGCCAGTGTTACAA CCAATTAACC	73STOP.FRT BAC cloning
FlpR_BglII	GATCAGATCTCCACCATGGCTCCTAAGAAG	pMSCV-Flp cloning
FlpR_EcoRI	GATCGAATTCTCATCAGATCCGCCTGTTGA	pMSCV-Flp cloning
73_IG_DS	GCCCTGGCGAAGGTGTTGCCCAGGATA	ORF73 PCR
73_IG_US	CCCACACCCTTCCTGTGCTAAAAGT	ORF73 PCR
59PCR1	ATGCAGACCTTCCAGCTTGAC	ORF59 PCR
59PCR2	CTCTTCCAAGGGAGCTTGCG	ORF59 PCR
