# Supplementary figures and images for: Conditional mutagenesis in vivo reveals cell type- and infection stage-specific requirements for LANA in chronic MHV68 infection
Source: PLoS Pathog. 2018 Jan 24;14(1):e1006865. doi: 10.1371/journal.ppat.1006865 (PMC5798852; doi:10.1371/journal.ppat.1006865)

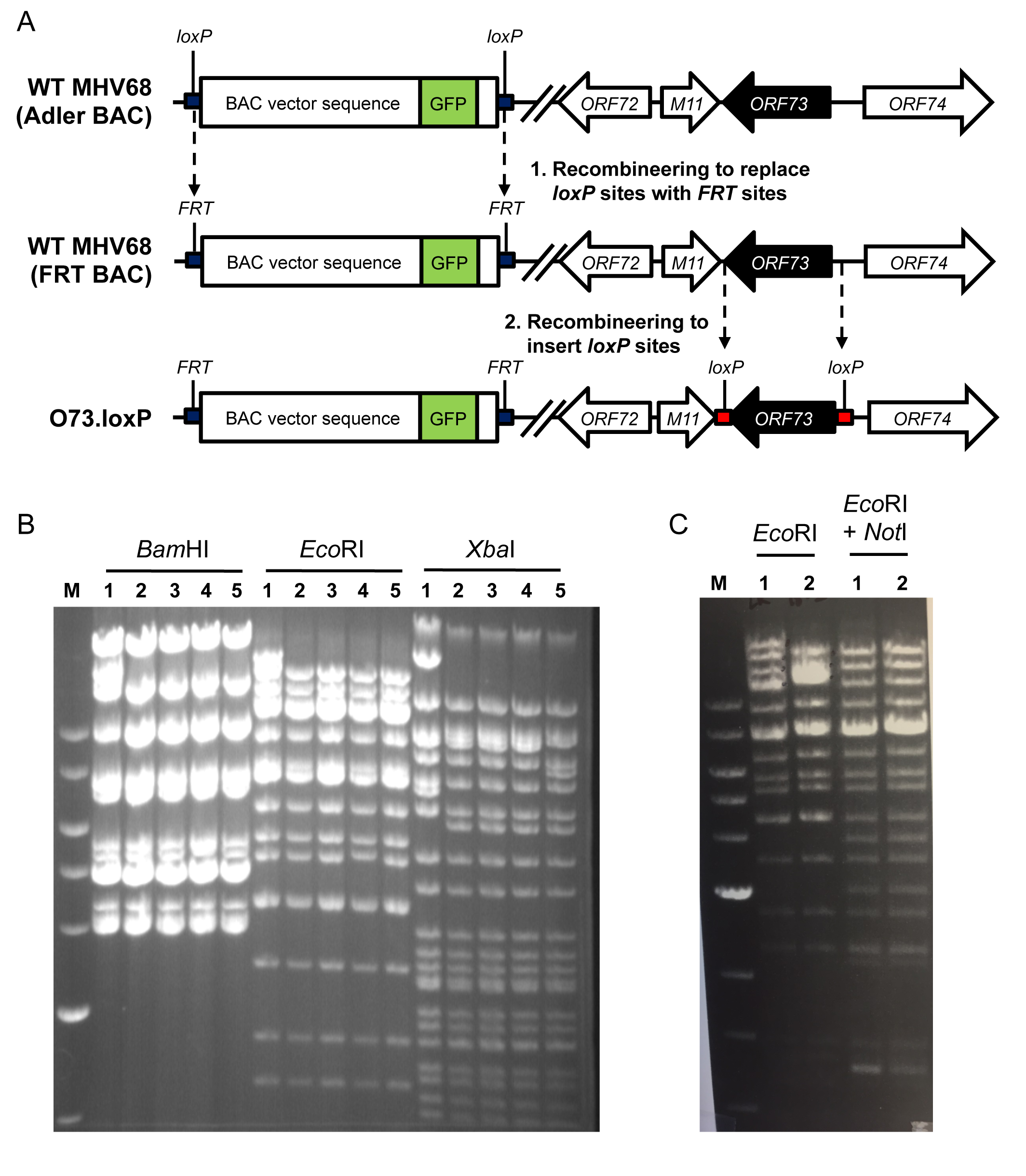

Supplement: S1 Fig — (A) Schematic depicting the strategy for modifying the MHV68 BAC by first replacing the loxP sites that flanked the BAC cassette with frt sites, followed by insertion of loxP sites that flanked ORF73. (B) En passant mutagenesis was performed on the parental MHV68 BAC (lane 1) to generate a new WT MHV68 BAC (FRT BAC, lanes 2 and 3). A subsequent round of en passant mutagenesis was performed on the FRT BAC to generate O73.loxP (lane 4) and 73.STOP (lane 5) BACs. BAC DNA was digested with the indicated restriction endonucleases, and digestion products were resolved by agarose-gel electrophoresis to evaluate the gross genetic integrity of the newly derived BACs. The mutation in 73.STOP generates a new XbaI site that results in a ~8 kb digestion product (see lane 5) not present in the other BACs. (C) The parental Adler (lane 1) and FRT (lane 2) BACs were digested with the indicated restriction endonucleases, and digestion products were resolved by gel eclectrophoresis. Although a larger than expected band is present for Adler BAC digested with EcoRI, the double digest that includes NotI, an enzyme that cuts once in each copy of the terminal repeat, results in an identical banding pattern for both BACs. This indicates that the large DNA fragments produced by single digests of the Adler BAC are due to the presence of more copies of the terminal repeats relative to FRT BAC constructs. (TIF) [file ppat.1006865.s002.tif]

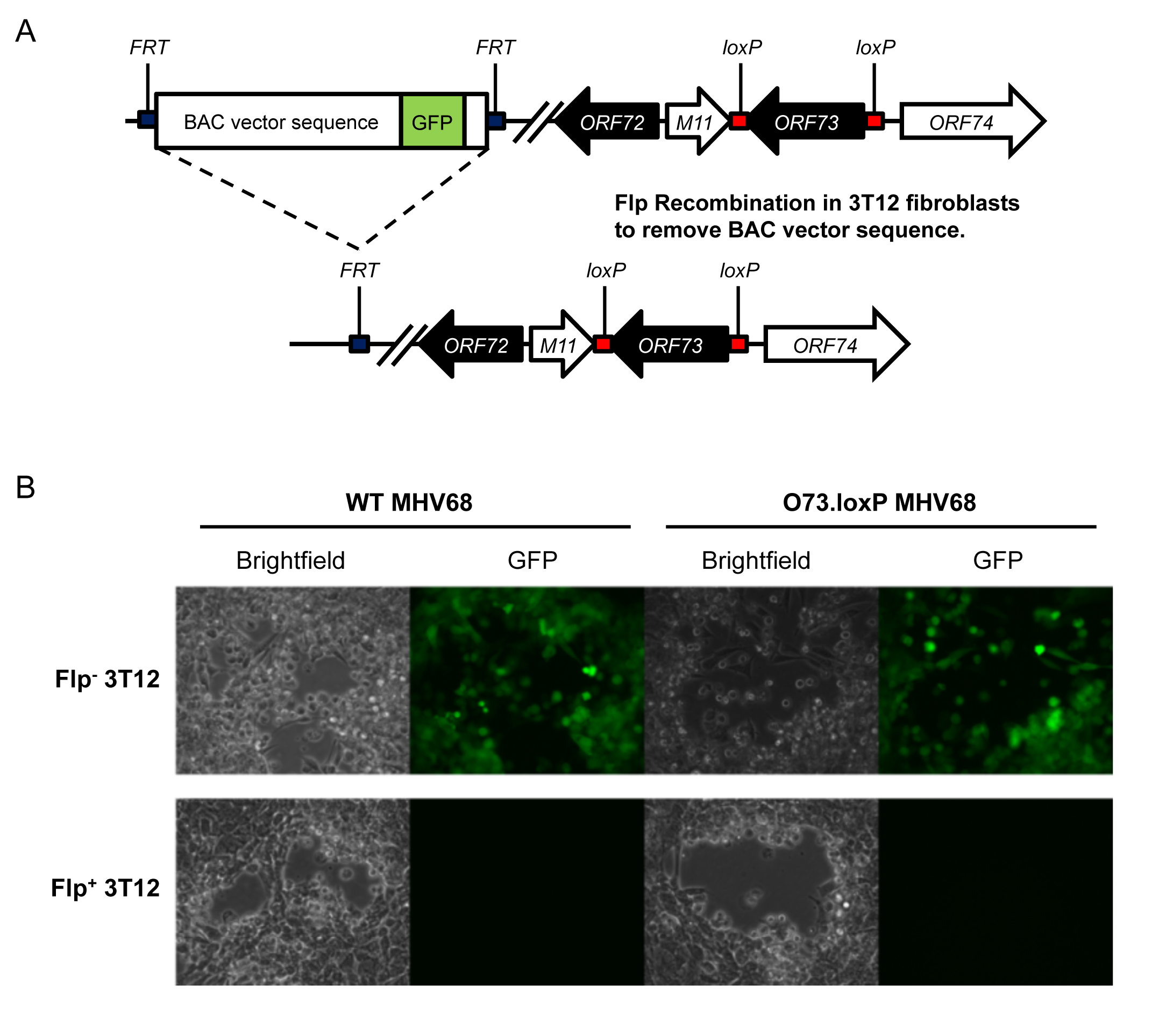

Supplement: S2 Fig — (A) Schematic depicting Flp-dependent removal of the BAC cassette, which encodes GFP, from the MHV68 genome. (B) 3T12 fibroblasts were stably transduced with empty vector or Flp recombinase encoding retroviruses and used to generate MHV68 stocks. For visualization and confirmation of BAC removal, cells were infected with WT MHV68 or O73.loxP grown in either Flp- or Flp+ cells at an MOI of 0.05 PFU/cell. Plaques were analyzed on day 4 post-infection by brightfield and fluorescence microscopy for the presence or absence of GFP. (TIF) [file ppat.1006865.s003.tif]

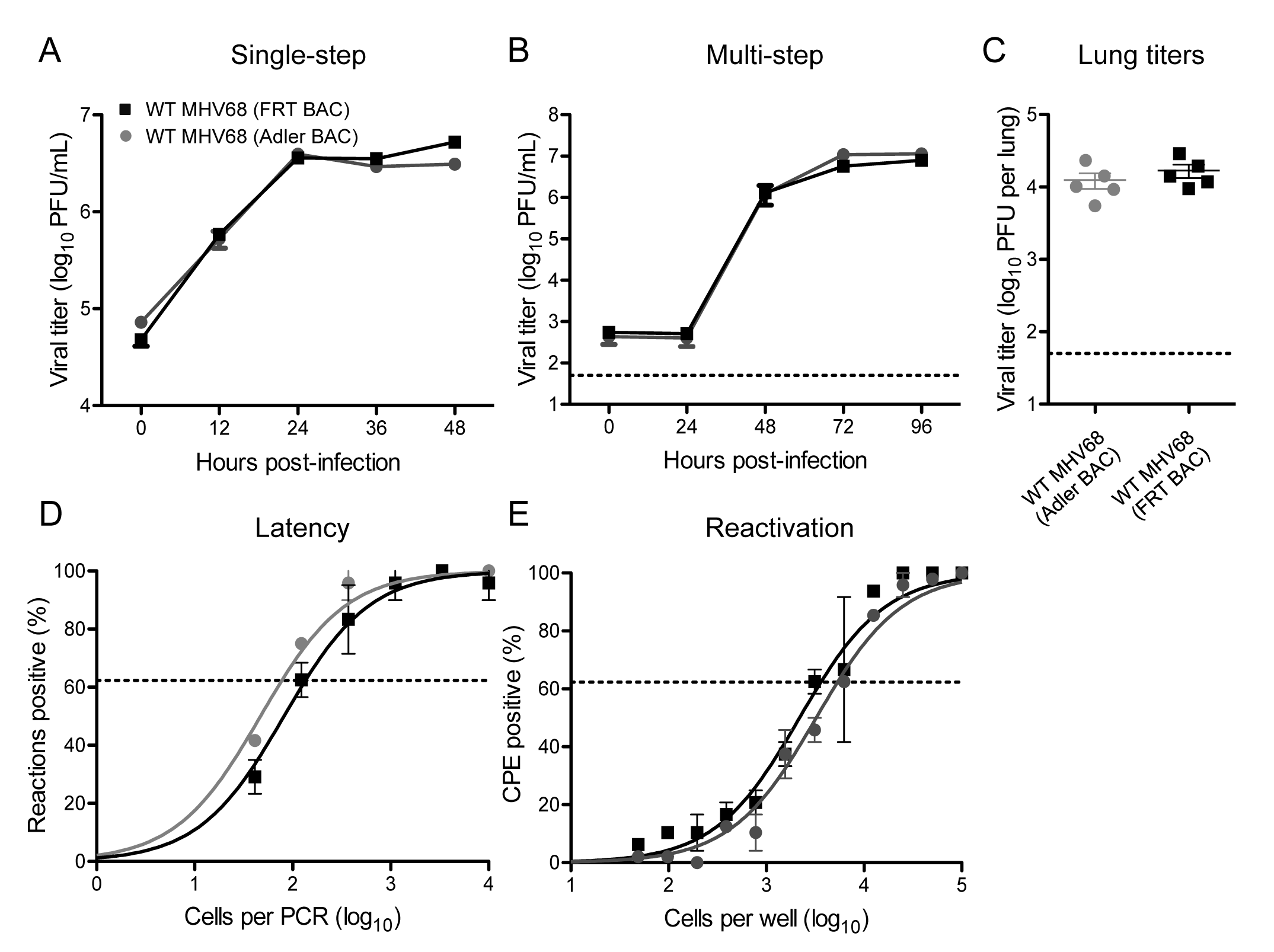

Supplement: S3 Fig — (A and B) 3T3 fibroblasts were infected with either Adler BAC- or FRT BAC-derived WT MHV68 at an MOI of 5 PFU/cell (single-step growth curve, A) or 0.05 PFU/cell (multi-step growth curve, B). Viral titers were determined at the indicated times post-infection by plaque assay. Results are means of triplicate samples. Error bars represent standard deviations. (C-E) C57BL/6 mice were infected IN with 1000 PFU of either Adler BAC- or FRT BAC-derived WT MHV68. (C) Mice were sacrificed on day 7 post-infection, and viral titers in lung homogenates were determined by plaque assay. Each dot represents one mouse. Error bars represent standard error of the means. (D and E) Mice were sacrificed on days 16–18 post-infection. (D) Single-cell suspensions of spleen cells were serially diluted and frequencies of cells harboring MHV68 genomes were determined using a limiting-dilution PCR analysis. (E) Reactivation frequencies were determined by ex vivo plating of serially diluted cells on an indicator monolayer. Cytopathic effect was scored 2–3 weeks post-plating. Groups of 3–5 mice were pooled for each infection and analysis. Results are means of three independent infections. Error bars represent standard error of the means. (TIF) [file ppat.1006865.s004.tif]

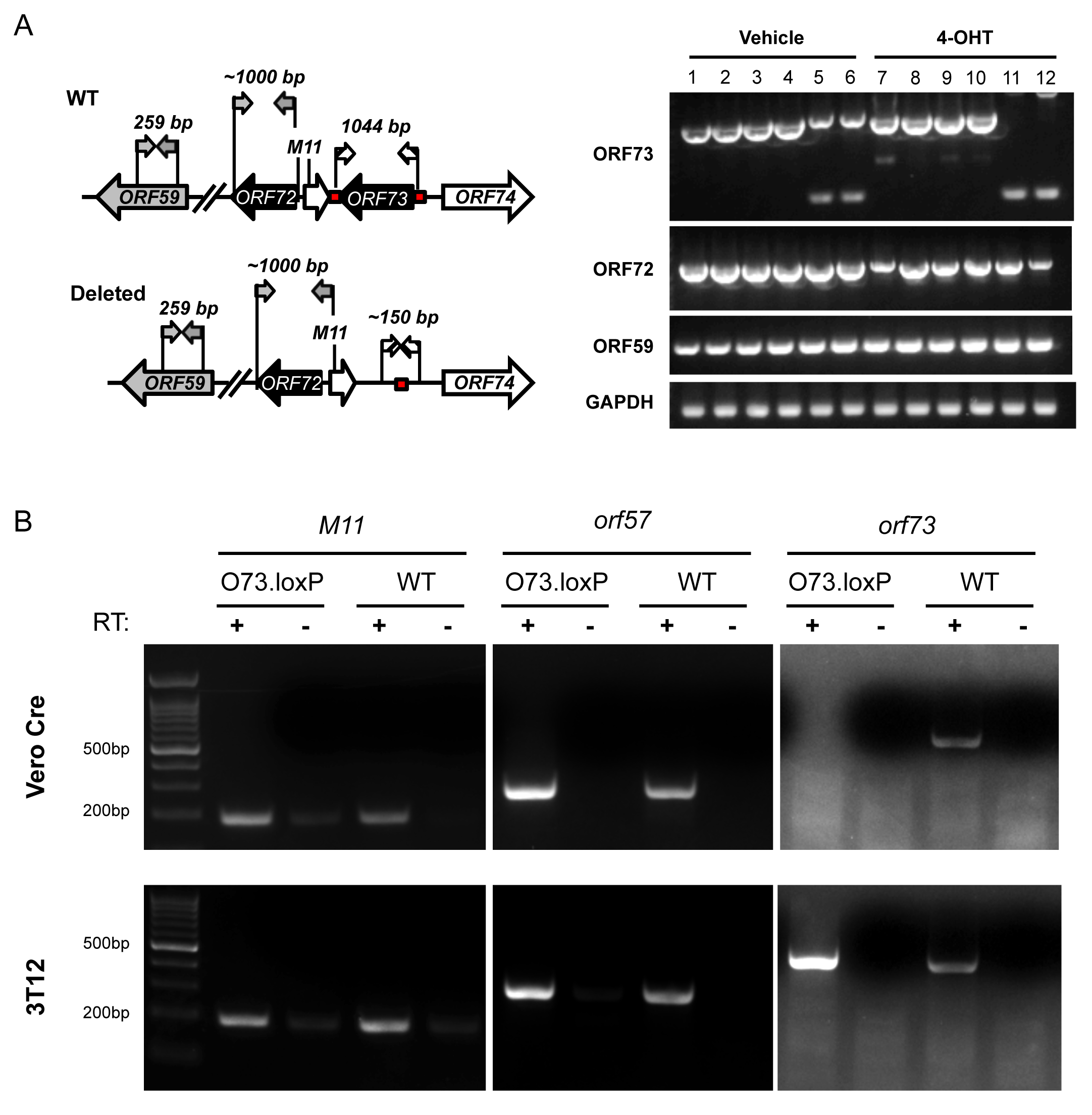

Supplement: S4 Fig — (A) 3T3 fibroblasts that encode Cre-ERT2 were treated with vehicle or 4-hydroxytamoxifen (4-OHT) to induce Cre activity 24 h prior to infection. Treated cells were infected with FRT BAC-derived WT MHV68 (isolate 1, lanes 3 and 9; isolate 2, lanes 4 and 10), O73.loxP (isolate 1; lanes 5 and 11; isolate 2, lanes 6 and 12), Adler BAC-derived WT MHV68 (lanes 1 and 7), or mLANA-null 73.STOP (lanes 2 and 8) at an MOI of 0.05 PFU/cell. Total DNA was isolated on day 4 post-infection, and PCR was performed as illustrated in the schematic to detect the indicated viral loci or cellular GAPDH as a control. (B) 3T12 fibroblasts or Vero cells constitutively expressing Cre recombinase were infected with WT MHV68 or O73.loxP MHV68 at an MOI of 0.1 PFU/cell. RNA was isolated on day 4 post-infection, and reverse transcription reactions were performed to with and without RT to generate cDNA. PCR was performed to detect the indicated viral transcripts. Products were resolved by agarose gel electrophoresis. (TIF) [file ppat.1006865.s005.tif]

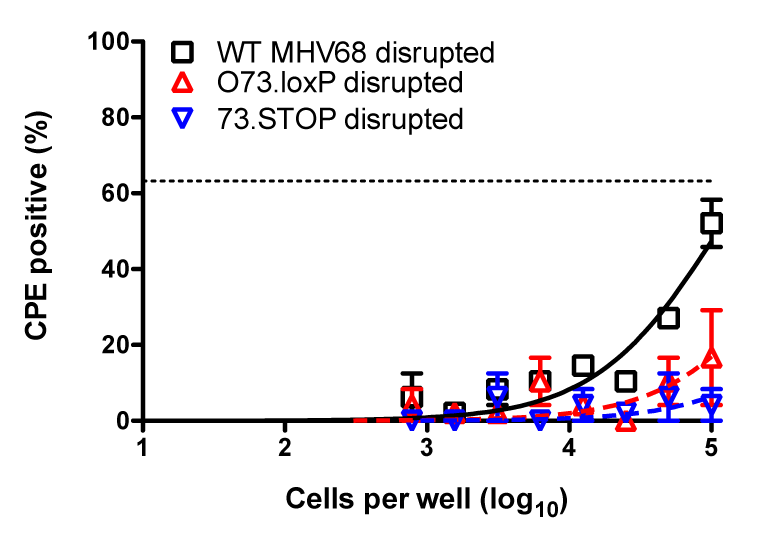

Supplement: S5 Fig — CD19Cre/+ mice were infected IN with 1000 PFU of the indicated viruses. Mice were sacrificed on day 10 post-infection and MLNs were harvested. Single-cell suspensions were subjected to hypotonic and mechanical lysis. Lysates were plated in a limiting-dilution manner on an indicator monolayer to quantify preformed infectious virus. Cytopathic effect was scored 2–3 weeks post-plating. Groups of 3–5 mice were pooled for each infection and analysis. Results are means of two independent infections. Error bars represent standard error of the means. (TIF) [file ppat.1006865.s006.tif]

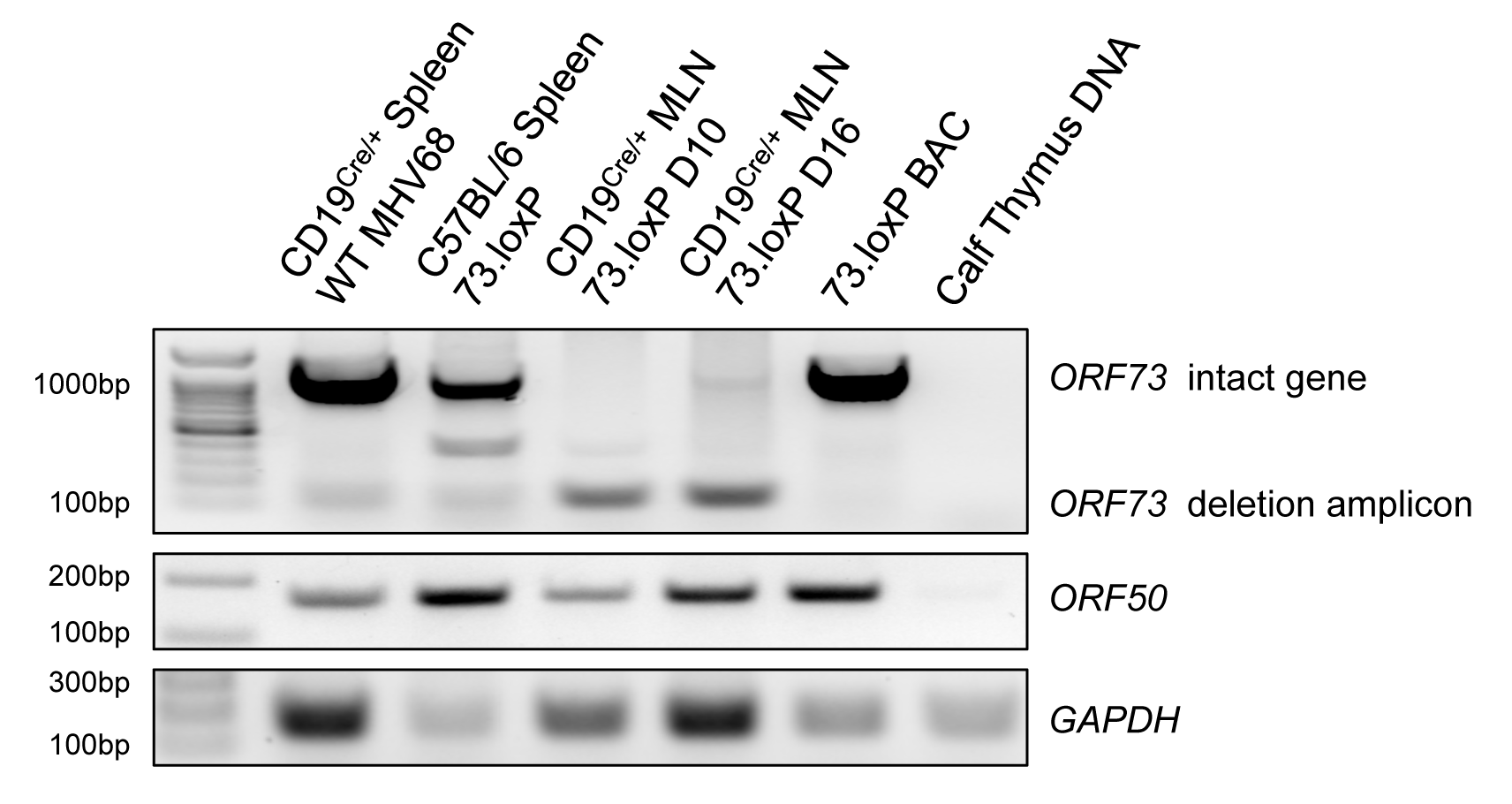

Supplement: S6 Fig — CD19Cre/+ mice were infected IN with 1000 PFU of O73.loxP MHV68. Mice were sacrificed on days 10 or 16 post-infection, and total DNA was isolated from mediastinal lymph nodes. PCR was performed to detect the indicated viral or cellular genes, and products were resolved by agarose gel electrophoresis. The additional samples represent comparative controls as a means to evaluate ORF73 deletion in the presence or absence of Cre recombinase. (TIF) [file ppat.1006865.s007.tif]

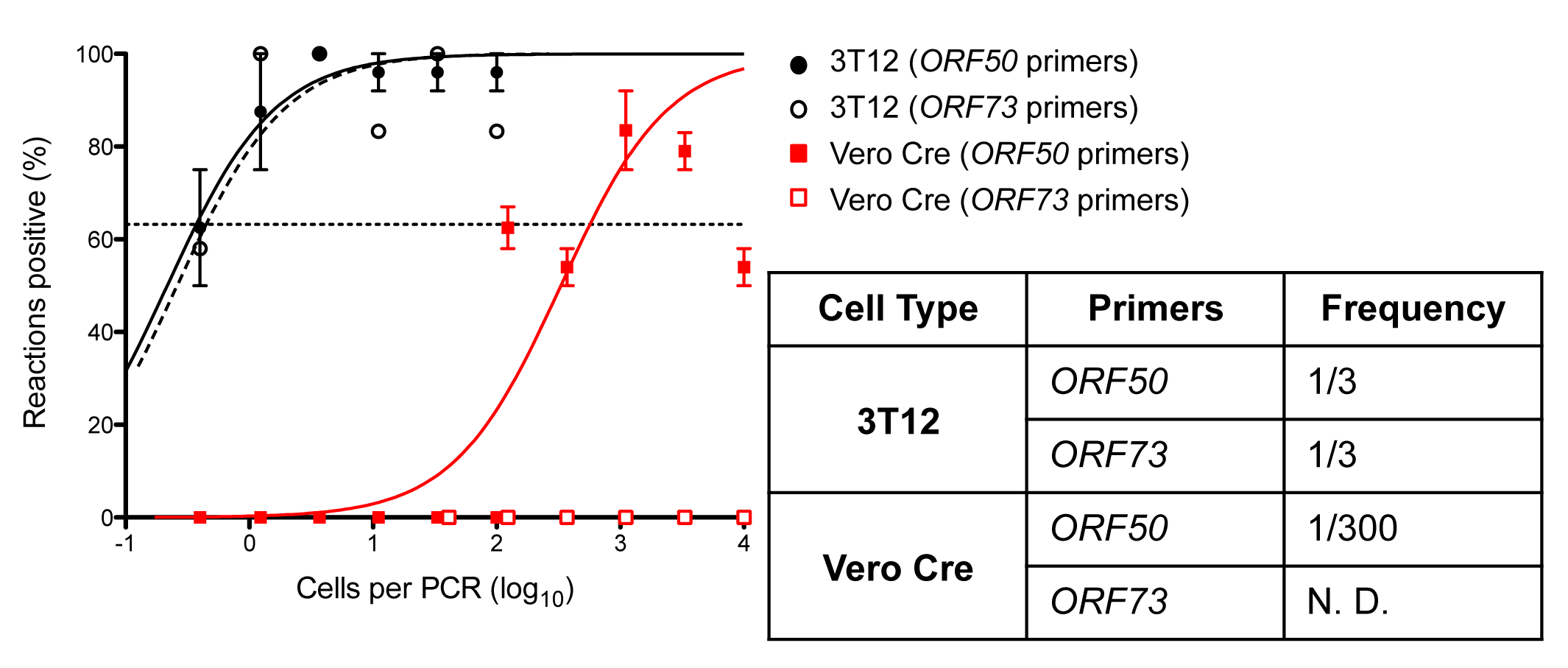

Supplement: S7 Fig — 3T12 fibroblasts or Vero cells constitutively expressing Cre recombinase were infected with WT MHV68 or O73.loxP MHV68 at an MOI of 0.1 PFU/cell. Cells were harvested on day 4 post-infection. Single-cell suspensions were serially diluted, and frequencies of cells harboring MHV68 genomes were determined using a limiting-dilution PCR analysis. In one set of analyses, primers specific for ORF50 were used. In another set of analyses, primers specific for ORF73 were used. Non-linear regression analyses were performed to determine the frequencies of cell harboring viral genomes. Viral genomes were equivalently detected by both primer sets in 3T12 fibroblasts lacking Cre. Viral genomes were detected with ORF50 primers, but not ORF73 primers, when cells expressing Cre were infected. Results are means of two independent experiments. Error bars represent standard error of the means. N.D. = not definable. (TIF) [file ppat.1006865.s008.tif]
